# Microbial origin of bioflocculation components within a promising natural bioflocculant resource of *Ruditapes philippinarum* conglutination mud from an aquaculture farm in Zhoushan, China

**DOI:** 10.1371/journal.pone.0217679

**Published:** 2019-06-19

**Authors:** Jun Mu, Xia Cui, Mingjiao Shao, Yuxia Wang, Qiao Yang, Guangfeng Yang, Liying Zheng

**Affiliations:** School of Marine Science and Technology, Zhejiang Ocean University, Zhoushan, Zhejiang, China; Columbia University, UNITED STATES

## Abstract

*Ruditapes philippinarum* conglutination mud (RPM) is a byproduct from the aquiculture of an important commercially bivalve mollusk *R*. *philippinarum* and has been recently reported as a promising natural bioflocculant resource. However the origin of bioflocculation components within RPM is still a pending doubt and impedes its effective exploitation. This study investigated the probability that RPM bioflocculation components originate from its associated microbes. RPM samples from an aquaculture farm in Zhoushan of China were applied to characterize its microbial community structure, screen associated bioflocculant-producing strains, and explore the homology between extracellular polysaccharides (EPS) from bioflocculant-producing isolates and RPM flocculation components. Results showed that RPM exhibited high bacterial biodiversity, with Proteobacteria, Bacteroidetes and Actinobacteria as the most abundant phyla; *hgcI_clade*, *CL500_29_marine_group*, *Fusibacter*, *MWH_UniP1_aquatic_group* and *Arcobacter* as the dominant genera. Fourteen highly efficient bioflocculant-producing strains were screened and phylogenetically identified as *Pseudoalteromonas* sp. (5), *Psychrobacter* sp. (3), *Halomonas* sp. (2), *Albirhodobacter* sp. (1), *Celeribacter* sp. (1), *Kocuria* sp. (1) and *Bacillus* sp. (1), all of which except *Bacillus* sp. were reported for the first time for their excellent flocculation capability. Furthermore, EPS from the bioflocculant-producing strains exhibited highly similar monosaccharide composition to the reported flocculation-effective RPM polysaccharides. On the other hand, the existence of fungi in RPM was rare and showed no flocculation functionality. Findings from Zhoushan RPM strongly supported that RPM flocculation components were of bacterial origin and make RPM reproduction possible by fermentation approach.

## Introduction

Microbial bioflocculants (MBF) mainly refer to extracellular polymer substances secreted by microorganisms during their special growth period and characteristic of harmlessness and biodegradability. There has been a history of exploiting bioflocculants from microorganisms of myriad habitats[1–5]. Although MBF are superior to traditional chemical flocculants due to their nontoxicity and environmental friendliness, high production costs and complicated fermentation/recovery processes have become the bottlenecks restraining their widespread commercial application, since most strains yield non-precipitating macromolecule polymers like extracellular polysaccharides (EPS) into the fermentation broth, and high-speed centrifugation and a large quantity of organic solvents are required for reclamation [6–8]. *Ruditapes philippinarum* conglutination mud (RPM), which refers particularly to the settled sludge from *R*. *philippinarum* when freshly harvested clams are kept in sterilized seawater for mud spitting, has been recently reported as a promising natural bioflocculant resource[9]. RPM possesses good flocculation capability, decoloration and heavy metal removal activity, especially exhibits easy recovery and fast settlement before and after treating pollutants[9–11].

Understanding the production mechanism of RPM bioflocculation components is critical to exploit and even reproduce this kind of bioflocculant resource. However, the origin of bioflocculation components within RPM is still a pending doubt. Previous study highlighted some clue that it may have relation with RPM associated microbes. However, till date, the microbial community profile in RPM is still not known and flocculant-producing microbial strains have been rarely screened and identified systematically[10, 11]. It is a necessity to characterize the microbial community structure of RPM and identify its associated bioflocculant-producing microbes. Additionally, since the flocculation-active polysaccharides of RPM have been recently reported for their composition[9], comparing the chemical composition of EPS of flocculant-producing microbial strains with RPM polysaccharides will give sound proof for judging the origin of RPM bioflocculation components.

In this study, with a purpose to clarify the probability that RPM bioflocculation components originate from its associated microbes, the microbial community structures of RPM from Zhoushan in China were characterized using high-throughput Miseq sequencing methodology; RPM associated bioflocculant-producing microbial strains were screened and phylogenetically identified, then their EPS composition were determined by HPLC and compared with RPM flocculation-active polysaccharides.

## Materials and methods

### RPM sample preparation

Fresh *R*. *philippinarum* clams were collected from an aquaculture farm (29°56′ N, 122°21′ E; permitted by Zhoushan Marine and Fisheries Bureau) located at zhujiajian island in Zhoushan, Zhejiang Province of China. The muddy clams were rinsed with sterilized seawater to remove surface sludge then carried back to the lab immediately. Clams (1 kg) were immersed into a tank with 2 L of sterilized seawater for 12 h at room temperature (around 25°C) for mud spitting, and settling mud samples from three replicates were obtained for high-throughput 16S rDNA Miseq sequencing.

### PCR amplicon and Illumina Miseq sequencing

Genomic DNA from RPM samples was extracted by the CTAB method [12]. Quality and quantity of DNA was verified with NanoDrop spectrophotometer (Nano Drop 2000, USA) and agarose gel. Extracted DNA was stored at -20 °C until further processing. For bacterial diversity analysis, the V3–V4 region of 16S rDNA was amplified to construct the bacterial community library, using the following prokaryotic primers: 343F (5'-TACGGRAGGCAGCAG-3'), and 798R (5'-AGGGTATCTAATCCT-3') [13].

50 ng DNA was used as the template for the polymerase chain reaction. The reaction was performed in a reaction system containing 15 μL of 2×*Taq* Master Mix DNA polymerase (Vazyme, Nanjing, China), 1 μL of forward and 1 μL of reverse primers, and 13 μL of H_2_O (including DNA solution volume). Template DNA was denatured at 94 °C for 5 min. The reaction was run for 25–35 cycles of 94 °C for 30 s, 60 °C for 30 s, and 72 °C for 30 s, and a final extension step of 72 °C for 7 min. Next, the PCR products were subjected to 1% agarose gel electrophoresis. Amplicon was purified with AMPure XP beads (Agencourt AMPure XP kit), and the amplicons were further amplified in another round of PCR. Primers Index i5 and Index i7 were used instead in the second round of PCR. 50 ng purified amplicon was added as the template. The same PCR conditions were applied except running for 6 cycles. After a second purification step with AMPure XP beads, the final amplicon was quantified using a Qubit dsDNA assay kit (Life Technologies), and the size of the final products was assessed with a Bioanalyzer 2100 (Agilent 2100, USA). Equal amounts of purified amplicon were pooled for subsequent Illumina Miseq sequencing.

Sequences obtained were preprocessed by Trimmomatic software to detect and cut off ambiguous bases [14]. After trimming, paired-end reads were assembled using FLASH software[15]. The sequences were then clustered into operational taxonomic units (OTUs) using UPARSE software, based on 97% similarity[16]. The representative read of each OTU was selected using the QIIME package[17]. Taxonomic classification of OTU representative sequences were subjected to a BLAST search against the Silva database Version 123 (https://www.arb-silva.de/download/archive/qiime/) using the RDP classifier (confidence threshold was 70%) [18]. The raw data of bacterial community was deposited in the NCBI database with an accession number of SRP128964.

### Isolation of target strains

Three fresh *R*. *philippinarum* clams (around 30 g) from the same site were placed in 90 mL of sterile seawater in a 200 mL Erlenmeyer flask, and they were kept spitting the activated mud for 12 h at room temperature (around 25 °C). One milliliter of thoroughly mixed liquid was pipetted out and diluted serially; the liquid was then spread onto isolation medium plates containing the following components (g·L^-1^): glucose, 20; (NH_4_)_2_SO_4,_ 0.2; urea, 0.5; yeast extract, 0.5; MgSO_4_·7H_2_O, 0.2; KH_2_PO_4_, 2.0; K_2_HPO_4_, 5.0; and agar, 20. The components were dissolved in 1 L artificial seawater (ASW), pH 7.2. The culture medium was sterilized at 115 °C for 30 min[10]. ASW contained the following components (g·L^-1^): CaCl_2_·2H_2_O, 1.36; MgCl_2_·6H_2_O, 9.68; KCl, 0.61; NaCl, 30.0; Na_2_HPO_4_, 0.014; Na_2_SO_4_, 3.47; NaHCO_3_, 0.17; KBr, 0.10; SrCl_2_·6H_2_O, 0.04; and H_3_BO_3,_ 0.03, pH 7.0. These components were dissolved in deionized water[19]. The inoculated plates were incubated for 30 d at 25 °C. The bacterial colonies with different morphology were selected successively during the long incubation period and purified by streaking. Pure strains were preserved on agar slants at 4 °C for further use.

Similarly, 1 mL of thoroughly mixed liquid was pipetted out and diluted serially; then, the liquid was spread onto two kinds of culture media suitable for fungal growth and isolation. One was the supplemented PDA culture medium containing the following components (g·L^-1^): potatoes, 4 (from 200 g infused potato); dextrose, 20; and agar, 20. These components were boiled and dissolved in 1 L of artificial seawater with an initial pH of 7.2. Penicillin and streptomycin were filter-sterilized into autoclaved medium to a final concentration of 60 mg·L^-1^ and 80 mg·L^-1^, respectively. The other was the Rose Bengal medium containing the following components (g·L^-1^): peptone, 5; glucose, 10; KH_2_PO_4_, 1.0; MgSO_4_, 0.5; chloramphenicol, 0.1, and agar, 20. The medium also contained 3.3 mL of 1% Rose Bengal solution; all of these components were dissolved in 1 L artificial seawater with an initial of pH 7.2. Both culture media were sterilized at 115 °C for 30 min. The inoculated plates were incubated for 30 d at 25 °C, followed by streaking purification.

### Screening for bioflocculant-producing microorganisms

Pure isolates were individually inoculated into 100 mL of liquid isolation medium (as described in isolation medium with agar omitted) and cultivated on a rotary shaker under shaking at 180 rpm at 25 °C for 5 d. The flocculation activity (calculated as flocculation rate, FR) of culture broth was measured using kaolin clay suspension method with slight modifications to the method[20]. Briefly, culture broth was centrifuged at 9000 × *g* for 10 min, and the supernatant was collected to determine FR. Ninety-three milliliters of kaolin clay suspension (4 g·L^-1^), 5 mL CaCl_2_ (10 g·L^-1^), and 2 mL supernatant sample were successively added in a 250-mL beaker, and the pH was adjusted to 7.5 with 2.0 mol/L sodium hydroxide solution or 2.0 mol/L hydrochloric acid. The mixture was vigorously stirred at 180 rpm for 1.0 min and slowly stirred at 60 rpm for 2.0 min; the mixture was then allowed to stand for 10 min. The optical density (OD value) of the clarifying surface layer was measured at 550 nm with a spectrophotometer (DR 1900–05, Hach, Shanghai, China). A control experiment with 2 mL deionized water, instead of the sample, was performed in parallel. The flocculation activity was calculated according to the following equation:

$$FR=\frac{A-B}{A}\times 100\%$$

where *FR* is the flocculation rate, and A and B are OD values of the control and sample, respectively. Each measurement was performed in triplicate, and the average value was calculated.

### Identification of bioflocculant-producing bacteria

The bacterial DNA extraction was performed using a genomic DNA extraction kit (TianGen, China), following the manufacturer’s protocol. The PCR amplification of 16S rDNA was performed using a PCR apparatus (GeneAmp 9700, ABI, USA). The 20-μL PCR reaction solution contained 0.8 μL of 5 μM primer 27F (5'-AGA GTT TGA TCM TGG CTCAG-3'), 0.8 μL of 5 μM primer 1492R (5'- T ACG GYT ACC TTG TTA CGA CTT-3') [21], 0.5 μL of 20ng/μL DNA template, 1.6 μL of 2.5 mM dNTP Mix, 2.0 μL of 10 × PCR buffer, 0.2 μL of 5 U/μL Ex *Taq* DNA polymerase (TaKaRa, Dalian, P. R. China), and 14.1 μL of double distilled H_2_O. The PCR reaction conditions involved denaturation of DNA at 95 °C for 5 min, followed by 30 cycles of denaturation at 95 °C for 30 s, annealing at 55 °C for 30 s, and extension at 72 °C for 90 s. The reaction mixture was further incubated at 72 °C for 10 min. Subsequently, the PCR products were purified on agarose gels and both strands were sequenced by a DNA Sequencer (3730XL, ABI, USA).

The 16S rDNA sequencing results were identified using EzBioCloud’s Identify service, which provides proven similarity-based searches against quality-controlled databases of 16S rDNA sequences (https://www.ezbiocloud.net/identify). MrBayes was used to construct the phylogenetic tree [22], based on 16S rDNA sequences of bioflocculant-producing bacteria and their top 10 closely related type strains from EzBioCloud’s Identify database. It was run for 1×10^5^ generations with a sampling frequency of 1000. For constructing phylogenetic trees, three heated chains were used at a heating temperature of 0.1. The 4by4 model was used with the GTR substitution model (nst = 6) and gamma-shaped rate variation (rates = invgamma). The phylogenetic tree was visualized using FigTree[23]. The 16S rDNA sequences of bioflocculant-producing bacteria were deposited in GenBank following NCBI accession Numbers: KX702255, KX702256, KX702257, KX702258, KX702259, KX702260, KX702261, KX702263, KX702264, KX702265, KX702266, KX702267, MG770447 and MG770448.

### EPS extraction and purification

The extraction and purification of EPS used the method of Choi et al. and Ugbenyen et al. with slight modifications[24, 25]. Bioflocculant-producing isolates were individually inoculated into 100 mL liquid isolation medium and cultivated at 180 rpm and 25 °C for 5 d. Then, culture broth was centrifuged at 9000 × g for 10 min (Thermofisher, USA) to remove cell pellets. The supernatant of each isolates was dialyzed in deionized water for 48 h and concentrated to 1/5 of the original volume using a rotary evaporator. The concentrated solution was mixed with three volumes of dehydrated cold ethanol and stayed overnight at 4 °C. The resulting precipitate was obtained by centrifugation at 6,000 × g for 20 min and lyophilization. After dissolving in distilled water, one volume of Sevag Reagent which was a mixed solution of trichloromethane and n-butylalcohol (5:2, V/V) was added to remove proteins. The uppermost aqueous phase was separated by centrifugation at 3000 × g for 10 min, added three volumes of dehydrated cold ethanol to precipitate and lyophilized again, then the purified EPS of isolates were obtained.

### EPS dosage-flocculation activity relation determination

EPS dosage-flocculation activity related experiments were performed respectively. 0–2.0 mg of EPS samples were dissolved into 2 mL deionized water and determined flocculation activity using method described in the section *screening for bioflocculant-producing microorganisms*.

### Chemical composition analysis of EPS from bioflocculant- producing isolates

HPLC analysis of EPS monosaccharide composition. The extracts of bioflocculant- producing isolates were analyzed using HPLC by the Dai’s method with slight modification[26]. For this analysis, 100 μL of polysaccharide sample solution with a concentration of 3 g·L^-1^ and 100 μL of 3 M trifluoroacetic acid (TFA) were pipetted into an ampoule, and hydrolyzed at 110°C for 2 h. After the hydrolyzed solution was cooled to room temperature, 200 μL of methanol was mixed and evaporated using the rotary evaporator to remove TFA. Then 50 μL of 0.3 M NaOH solution was added to the evaporated sample to dissolve the residue, and derivatized with 50 μL (0.5 M) of PMP methanolic solution in a small sample tube at 70 °C for 100 min. The derivatized mixture was cooled to room temperature and neutralized with 50 μL of 0.3 M HCl, then added water to replenish the volume to 1 mL. The reaction mixture was extracted by adding an equal volume of chloroform, discarded the chloroform phase and repeated this extraction process three times. The extracted liquid was filtered through a 0.45 μm microporous membrane for subsequent HPLC sample analysis. All the purified samples of bioflocculant-producing isolates and the mixed monosaccharide standard sample were subjected to the same hydrolysis and derivatization steps as described previously. Agilent 1100 HPLC system (USA) with a photodiode array detector was used for chromatographic analysis of samples and the analysis conditions as follows: column, ZORBAX Eclipse XDB-C18, 250 mm × 4.6 mm, 5 μm (Agilent Technologies, USA); mobile phase, 0.1 M phosphate buffer (pH 6.7) and acetonitrile (83:17, v/v%);column oven temperature, 30 °C; detection wavelength, 254 nm; flow rate, 0.9 mL·min^-1^, injection volume, 20 μL.

Monosaccharides (D-glucosamine, D-galactose, D-arabinose, D-xylose, D-galactosamine, D-glucuronic acid, L-fucose, D-ribose, D-galacturonic acid) were purchased from Shanghai Yuanye Biotech. Co. (China). The other standard monosaccharides (D-mannose, D-glucose and L-rhamnose) were obtained from Dr. Ehrenstorfer GmbH (Germany). 1-Phenyl-3-methyl-5-pyrazolone (PMP) and HPLC-grade acetonitrile were purchased from USA Tedia Co.

## Results

### Microbial community composition by Illumina MiSeq sequencing

**Fig 1:**
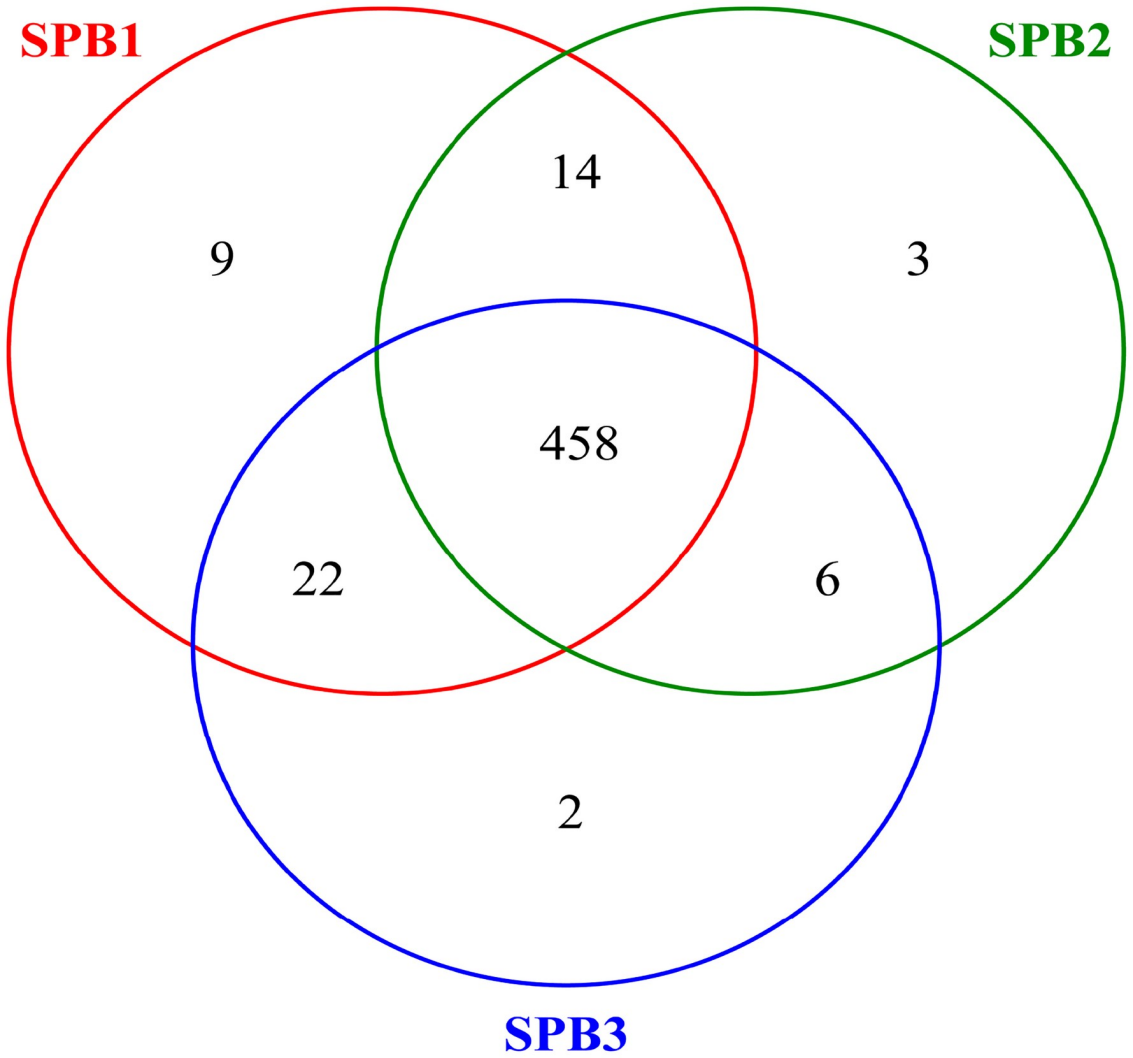
Venn plot showing the common and individual OTUs of the RPM samples.

The total number of effective bacterial sequences in RPM was 90,963 with an average length of 421.8 bp, as determined by Illumina MiSeq sequencing. The high-quality 16S rDNA sequences were clustered into 514 OTUs with at least 97% similarity. The taxonomical classification and abundance of all OTUs are shown in S1 Table. The rarefaction curves in S1 Fig suggested reasonable sequencing depth and Good’s coverage of 0.9992–0.9994, as reported in Table 1 was in agreement with this data. The alpha diversity of three parallel samples of RPM gave rather similar results (Table 1). OTUs from SPB1, SPB2, and SPB3 ranged from 481–502, with a sharing part of 458 (Fig 1). Chao1 richness was calculated to be between 489.8–510. Shannon Wiener and Simpson indices were quite high relative to the observed OTU numbers, for which could be expressed as Pielou evenness index with a value of 0.80–0.81, indicating a high biodiversity.

**Table 1:** Estimation of bacterial richness and diversity of the three RPM samples based on MiSeq sequencing.

Sample	OTUs	Chao1	Shannon Wiener	Simpson	Good’s Coverage
SPB1	502	510	7.18	0.98	0.9994
SPB2	481	489.8	7.23	0.98	0.9992
SPB3	487	496.7	7.27	0.98	0.9994

**Fig 2:**
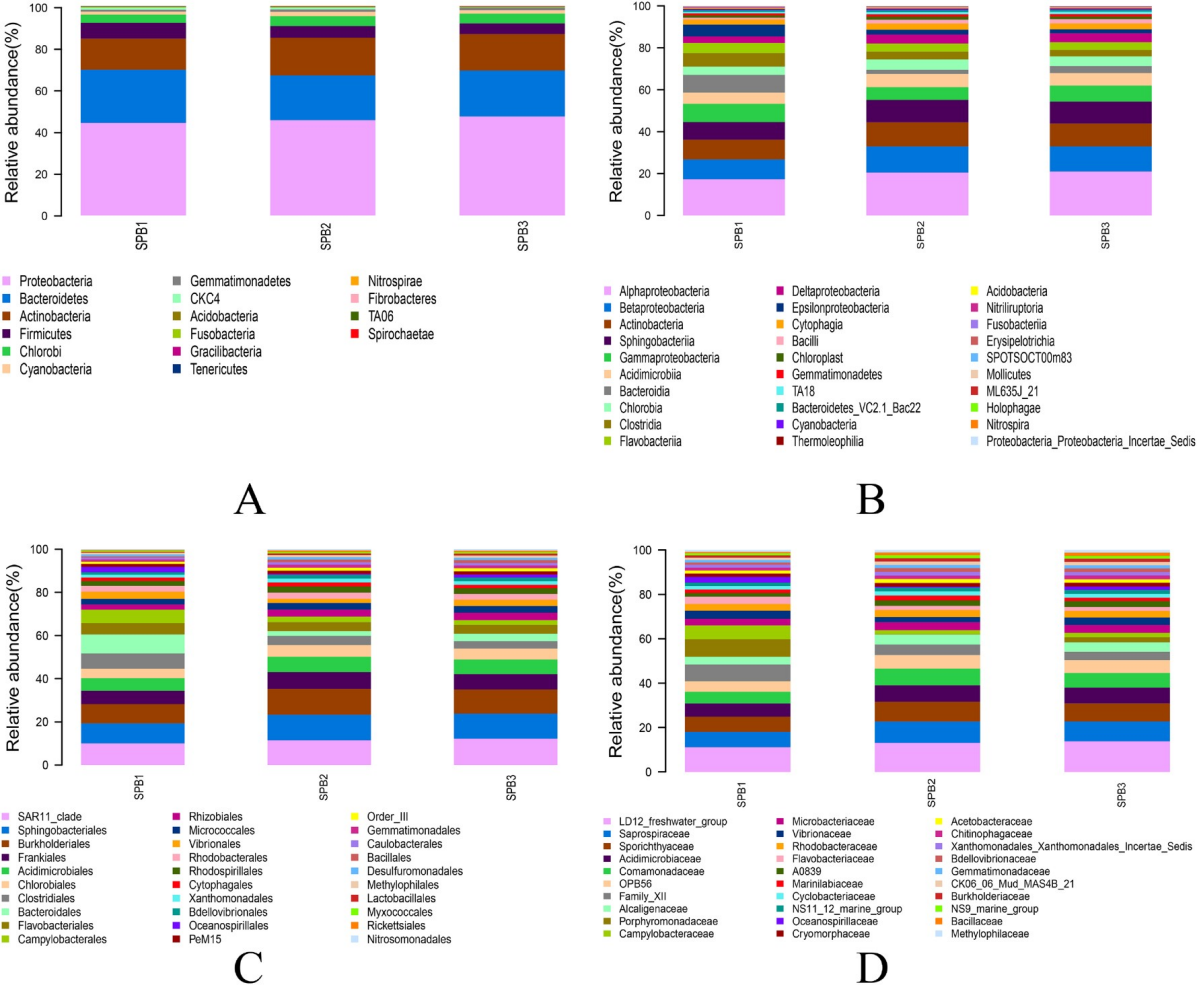
Relative abundance of top 30 bacterial phyla (A), classes (B), orders (C), and families (D) in RPM communities by 16S rDNA sequencing.

The relative abundance of the bacterial community in the three RPM samples was analyzed based on the results of OTU taxonomy classification. The samples harbored 16 phyla and 32 classes via BLAST. Fig 2A showed that Proteobacteria (46%) was the most abundant phylum in the three samples, followed by Bacteroidetes (23%) and Actinobacteria (17%), all of which accounted for more than 86% of the total sequences. For Proteobacteria, Alphaproteobacteria (19%), Betaproteobacteria (11%), and Gammaproteobacteria (7%) were the major classes, while Sphingobacteriia (10%) and Bacteroidia (5%) represented the most abundant classes within Bacteroidetes; Actinobacteria (10%) and Acidimicrobiia (6%) dominated the Actinobacteria phylum (Fig 2B).

Sequences belonging to a total of 78 bacterial orders and 132 families were identified. SAR11_clade (10%) in Alphaproteobacteria, Sphingobacteriales (10%) in Sphingobacteriia, Burkholderiales (9%) in Betaproteobacteria, Frankiales (6%) in Actinobacteria, and Acidimicrobiales (6%) in Acidimicrobiia were the representative orders in RPM samples, (Fig 2C). The SAR11_clade was represented by the family LD12_freshwater_group (10%), while Sphingobacteriales was represented by the family Saprospiraceae (7%), and Frankiales by Sporichthyaceae (6%) (Fig 2D).

**Fig 3:**
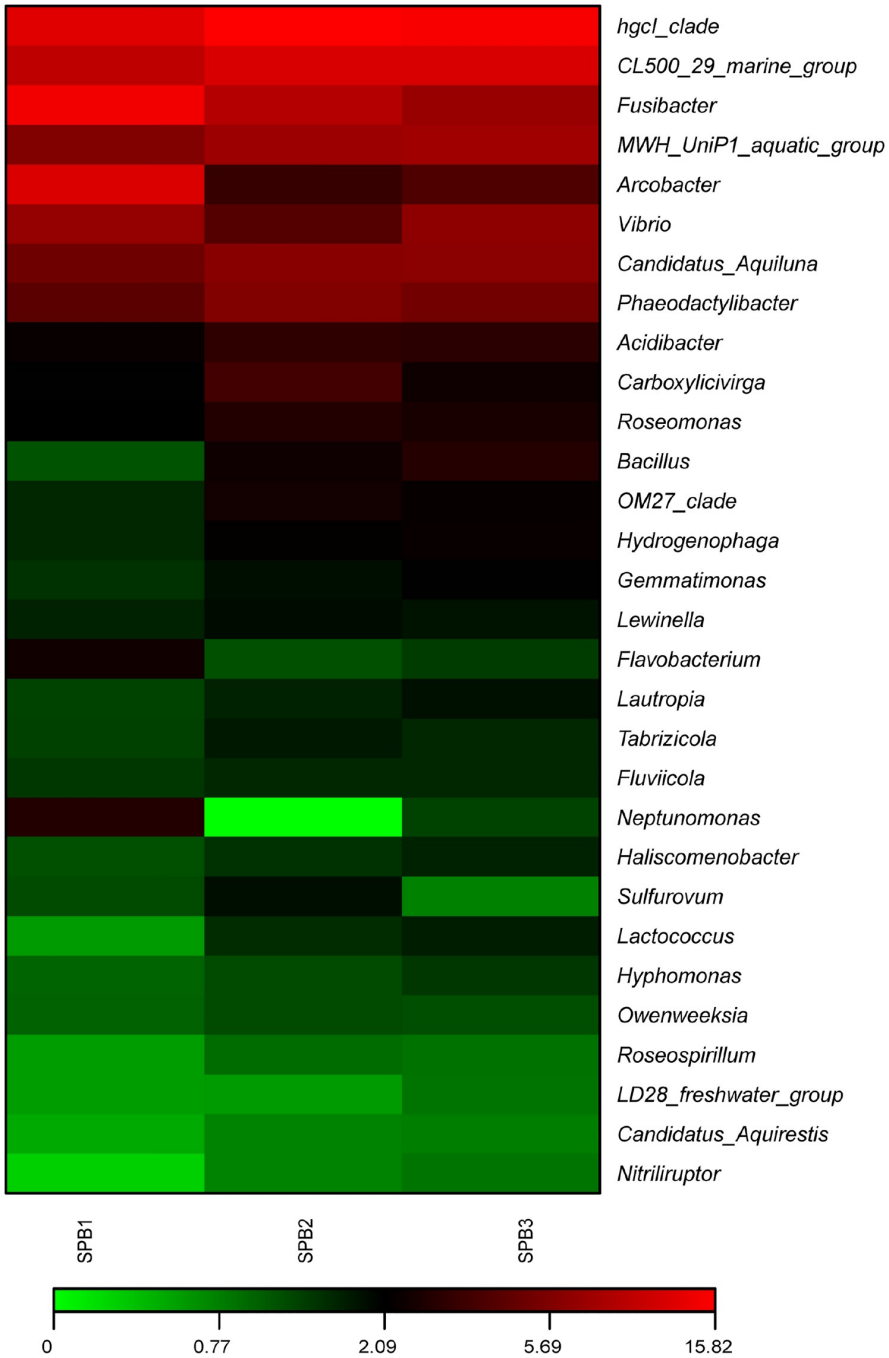
Heat-map representing the relative abundance of top 30 genera in three parallel RPM samples, SPB1, SPB2, and SPB3. The gradient colors from green to red represent increasing relative abundance.

As shown in Fig 3, 139 different taxa were classified at the level of genus; the top five genera included *hgcI_clade*, *CL500_29_marine_group*, *Fusibacter*, *MWH_UniP1_aquatic_group*, and *Arcobacter*, which, in total, accounted for more than 20% of all the classified sequences.

In contrast, high throughput sequencing of fungal sequences could not be carried out since very low fungal DNA content in RPM led to the unsuccessful PCR amplification of the ITS1 region, which was critical for effective Illumina Miseq sequencing.

### Bioflocculant-producing microorganism screening and phylogenetic identification

**Fig 4:**
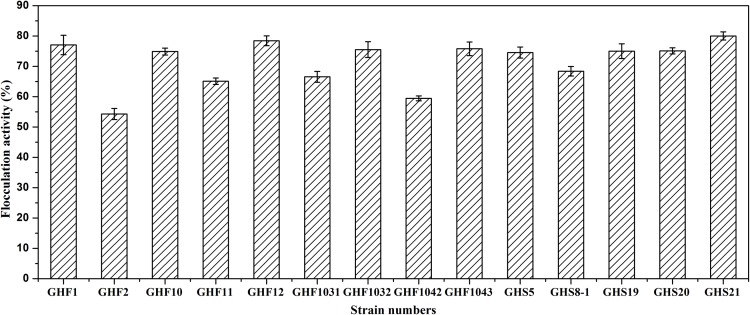
Flocculation activity of fermentation liquor from bioflocculant-producing bacteria.

A total of 14 bioflocculant-producing bacterial strains were screened from 32 diverse colony morphological phenotypes isolated from RPM. Fig 4 shows the bioactive strains obtained, wherein the highest FR of 80% was observed for GHS21, while FR of over 70% was observed for 8 strains (GHF1, GHF10, GHF1032, GHF1043, GHF12, GHS5, GHS19, and GHS20); 4 strains including GHF11, GHF1031, GHF1042, and GHS8-1 showed FR of more than 60%, while GHF2 exhibited a relatively lower FR of 53.8%. Compared with the 14 bioflocculant-producing bacterial strains, the other isolates from RPM showed much lower FR ranging from 0% to 30% (negative control). A high bioflocculation-active bacterial strain ZHT4-13 could serve as a positive control with a FR of 83.9% [10].

**Fig 5:**
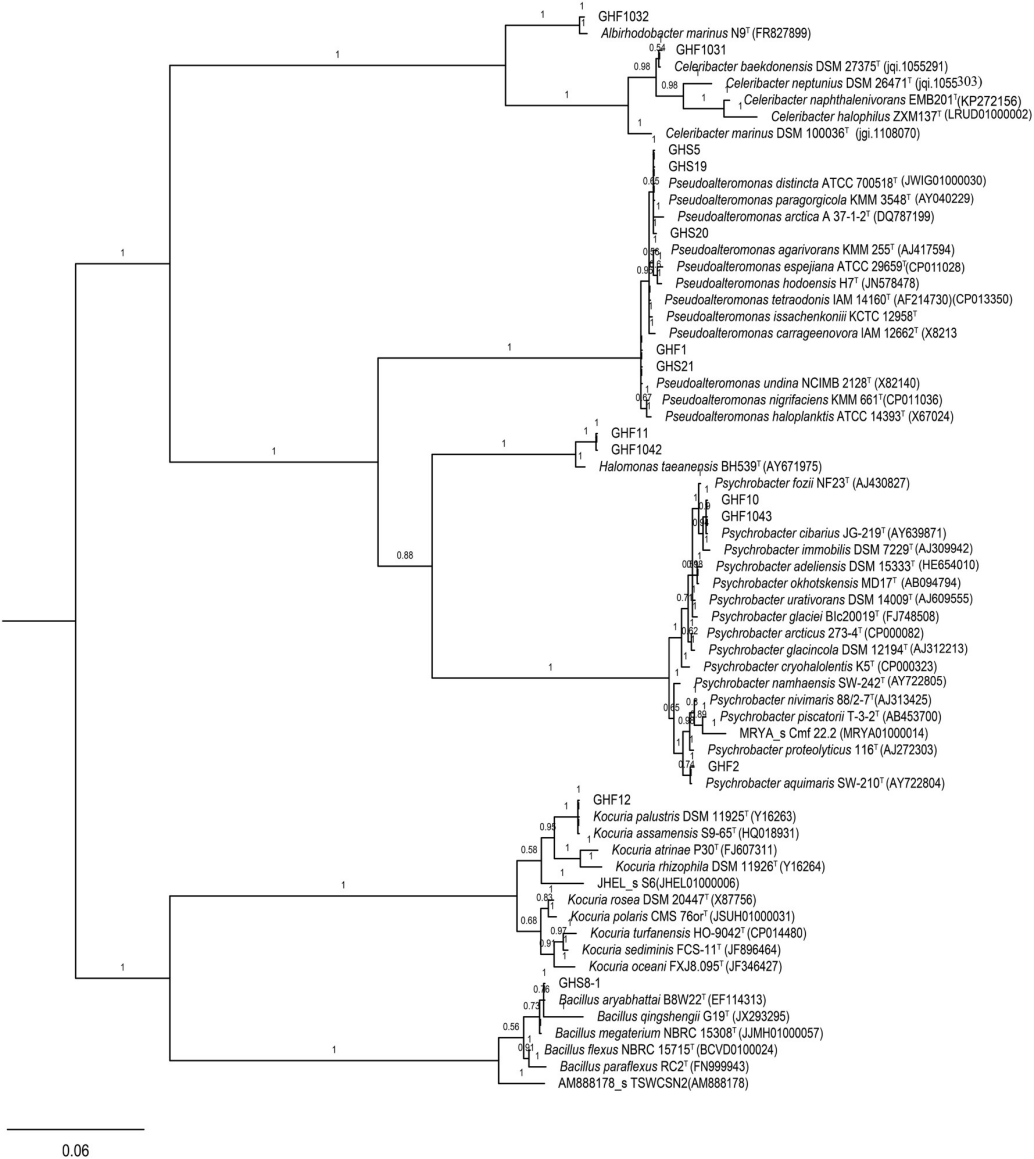
The phylogenetic relationship of the 14 bioflocculant-producing isolates and their top 10 closely related type strains identified in the EzBioCloud’s database. The posterior probabilities are shown on branch labels and calculated by MrBayes.

The 16S rDNA sequence-based identification was performed using EzBioCloud’s Identify service. All bioflocculant-producing isolates could find their respective counterpart type strains with the highest 16S rDNA sequence similarity of 98.83%–100% in EzBioCloud’s database (S2 Table). The phylogenetic relationship of the bioflocculant-producing isolates and their top 10 closely related type strains from EzBioCloud’s Identify database are shown in Fig 5. Among the bioflocculant-producing bacteria, 10 of the 14 isolates belonged to Gammaproteobacteria, namely, *Pseudoalteromonas* sp. (GHF1, GHS5, GHS19, GHS20, and GHS21), *Psychrobacter* sp. (GHF2, GHF10, and GHF1043), and *Halomonas* sp. (GHF1042 and GHF11); 2 isolates were classified as Alphaproteobacteria, namely, *Albirhodobacter* sp. (GHF1032) and *Celeribacter* sp. (GHF1031); GHF12 was identified to be a *Kocuria* sp. and GHS8-1 to be a *Bacillus* sp.

During fungal isolation and screening, yeast or mold colonies could hardly be obtained using the marine PDA medium and marine Rose Bengal medium. The only 3 yeast-like colonies obtained from spreading PDA plates with 1 mL raw RPM suspension gave negative results during the subsequent flocculation activity screening.

### EPS dosage-flocculation activity

GHF1, GHF1042, GHS20 and GHS21 represented relatively higher polysaccharide-yielding strains thus were selected for EPS dosage-flocculation activity relation determination. EPS from strains GHF1, GHF1042, GHS20 and GHS21 exhibited FR of 51%, 49%, 53% and 55% at dosages of 0.3mg, 0.12mg, 0.05mg and 0.2mg respectively. These results confirmed that EPS produced by those bioflocculation-active strains are effective bioflocculants.

### EPS monosaccharide composition of bioflocculant-producing isolates

**Fig 6:**
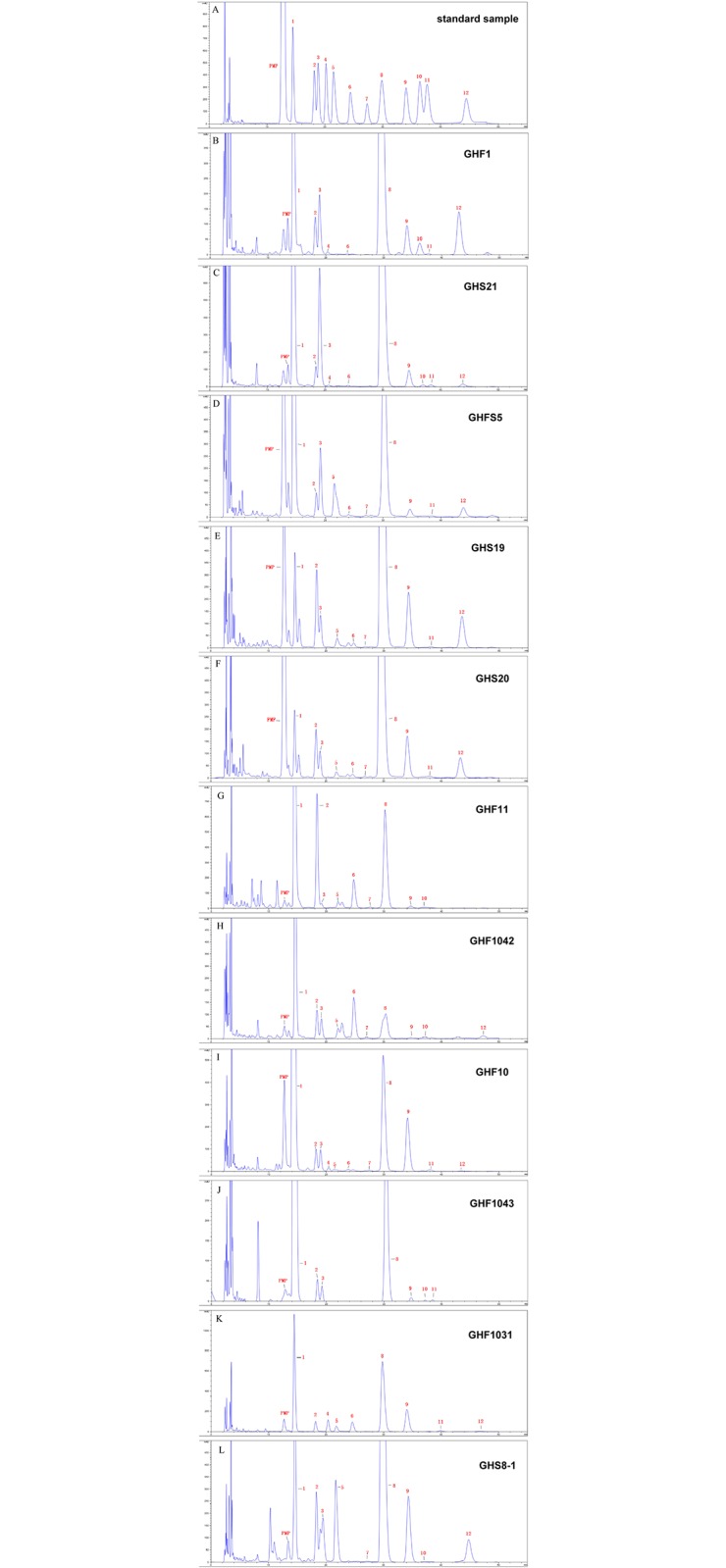
High-performance liquid chromatograms of PMP pre-column derivatives of monosaccharide standard sample (A) and hydrolyzed monosaccharides derivatives from isolates (B-L). Peaks:Man (1); GlcN (2); Rib (3); Rha (4); GlcUA (5); GalUA (6); GalN (7); Glc (8); Gal (9); Xyl (10); Ara (11); Fuc (12).

**Fig 7:**
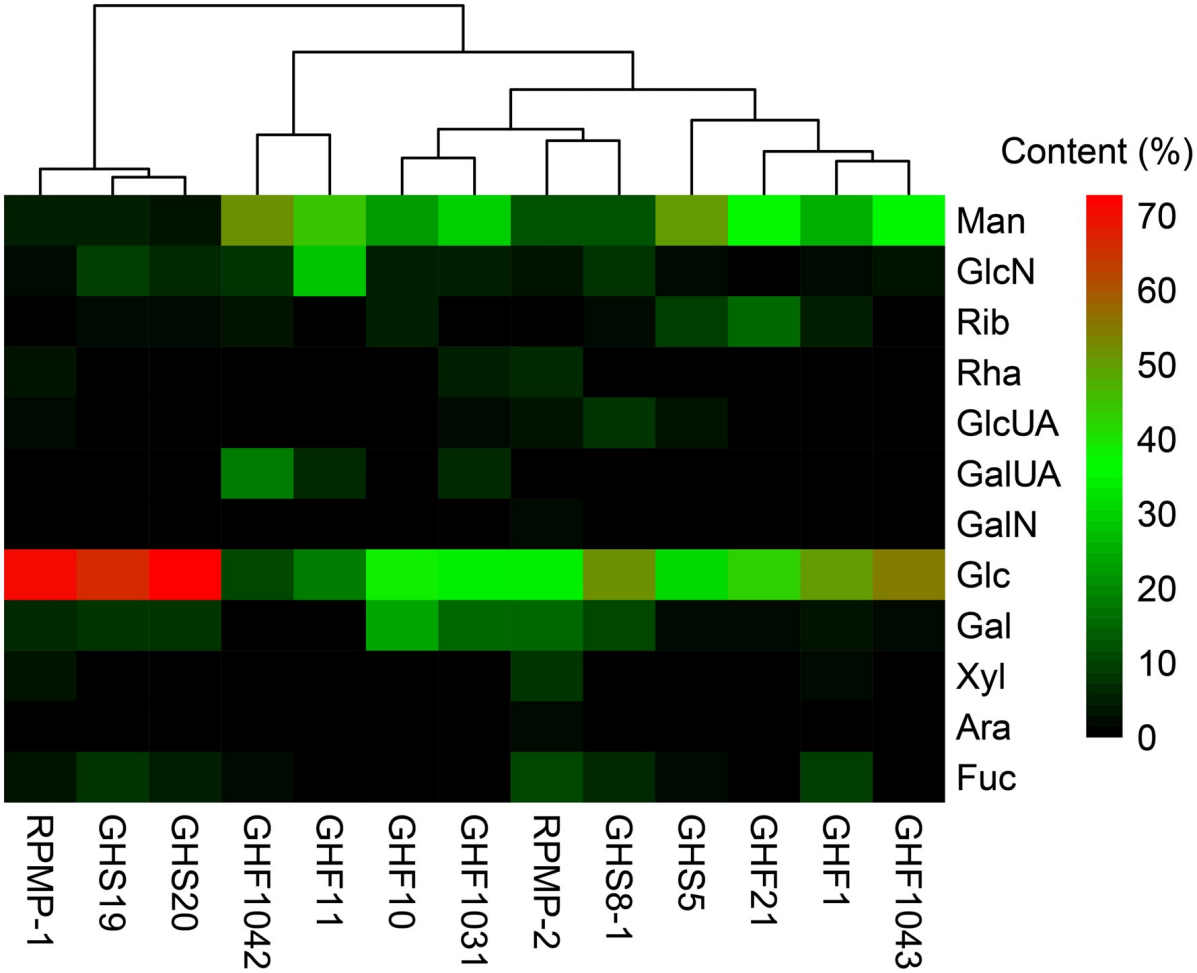
Monosaccharide composition Heat-map of EPS from bioflocculant-producing isolates and flocculation-active polysaccharides RPMP-1 and RPMP-2 from RPM. Heat-map was constructed and the gradient colors represent corresponding monosaccharide content (%). Man, D-mannose; GlcN, D-galactosamine; Rib, D-ribose; Rha, L-rhamnose; GlcUA, D-glucuronic acid; GalUA, D-galacturonic acid; GalN, D-glucosamine; Glc, D-glucose; Gal, D-galactose; Xyl, D-xylose; Ara, D-arabinose; Fuc, L-fucose.

EPS from eleven bioflocculant-producing bacterial isolates were successfully determined, all of which were complex heteropolysaccharides. Their respective HPLC chromotographs are shown in Fig 6. Twelve kinds of monosaccharides occurred with a varied proportion in these strains, including Man, GlcN, Rib, Rha, GlcUA, GalUA, GalN, Glc, Gal, Xyl, Ara and Fuc (S3 Table). A Heat-map as Fig 7 was constructed for comparing monosaccharide composition of EPS from bioflocculant-producing isolates with flocculation-active polysaccharides RPMP-1 and RPMP-2 from RPM[9]. It’s shown that RPMP-1 was highly similar in monosaccharide composition to GHS19 and GHS20, while RPMP-2 was similar in monosaccharide composition to GHS8-1, though both of them still shared common monosaccharide types with the other strains. Up to date, EPS from the collection of RPM-associated bioflocculant-producing isolates showed no similarity with reported microbial bioflocculants for their complicated composition. As one of negative controls, a bioflocculant produced by *Bacillus megaterium* consisted of glucose and mannose with the mole ration of 4:1, which is quite different from RPM-associated microbial EPS [27].

## Discussion

*R*. *philippinarum* is a filter feeding bivalve which takes in water that has plankton floating in it[28]. With two typical siphons (one in and one out), a large quantity of microorganisms is sucked in during the feeding process and simultaneously wastes are expelled out as RPM. RPM comprises not only cell debris, but also live microbiota which reflects the characteristic endogenous microcosm of *R*. *philippinarum*. In our previous studies, RPM was discovered to be a promising natural bioflocculant resource showing excellent flocculation and decoloration activities[9, 11]. Naturally collected RPM exhibits high flocculation activities to kaolin clay both in deionized water and sea water assay systems, and also could flocculate marine microalgae[9]. Further research triggered speculation that bioflocculant activities of RPM might be attributed to *R*. *philippinarum*-associated microbiota. An experiment was performed using antibacterial and antifungal antibiotics to selectively inhibit bacteria or fungi in the temporary cultivation system of *R*. *philippinarum*. The result showed that RPM from antibacterial temporary cultivation group exhibited a significant decrease in flocculation activity, while RPM from the antifungal selectively inhibited group still kept a high flocculation activity similar to a normal cultivation group, which indirectly suggested that bacteria may be the key factor in the flocculation activity development of RPM [29]. However, more comprehensive and direct proofs were still needed to support the hypothesis of RPM flocculation origin from microorganisms. This study combined non-culturable, culturable and microbial EPS analytical approaches to verify that the RPM associated microbes played a key role in RPM flocculation.

Illumina Miseq sequencing as the non-culturable approach could be used to examine the microbiome constitution of RPM. However, it is difficult to distinguish live bacteria from dead cells, including cell debris, with the remaining effective 16S rDNA fragments by current high throughput sequencing methods. Thus, species detected by Illumina Miseq sequencing may exceed live biota species in RPM. At the genus level, dead cells from filter feeding increased the diversity of RPM and decreased the abundance of the other biota. This might partly explain that as many as 139 genera were identified and the top five genera, including *hgcI_clade*, *CL500_29_marine_group*, *Fusibacter*, *MWH_UniP1_aquatic_group*, and *Arcobacter*, only occupied more than 20% in all classified sequences. Within the first abundant five genera, *hgcI_clade*, *CL500_29_marine_group*, and *MWH_UniP1_aquatic_group* are bacterial genera that cannot be cultured at present and are found in a variety of freshwater or marine environments[30–32].

In the course of fungal community analysis and bioflocculant-producing strain screening from RPM, the isolation approach yielded result essentially in agreement with that of the Illumina Miseq sequencing attempt, since the scarce existence of fungi in RPM led to inefficient ITS amplification and poor fungal growth on both marine PDA and marine Rose Bengal media plates. Zhou et al. [29] confirmed that fungi did not play a role in the bioflocculation functionality of RPM, since upon the addition of antifungal antibiotics in the temporary cultivation group of *R*. *philippinarum*, no remarkable decrease in flocculation activity of RPM was observed. Therefore, it can be tentatively concluded that fungi are rarely present in RPM and do not possess flocculation functionality.

Regarding the respective counterpart type strains with the highest 16S rDNA sequence similarity of bioflocculant-producing isolates from RPM, all of them except *Bacillus* sp. were reported for the first time that held excellent flocculation capability; moreover, most of their corresponding type species have been obtained from brackish or seawater surroundings. Strains GHS5, GHS19 and GHS20 all showed 100% 16s rDNA similarity to *Pseudoalteromonas paragorgicola and Pseudoalteromonas distincta*. *P*. *paragorgicola* could grow at NaCl concentrations of 1–8% [33] and *P*. *distincta* was isolated from marine sponge[34]. Strain GHF12 exhibited 100% 16s rDNA identity to *Kocuria palustris* and *Kocuria*. *assamensis*. *K*. *palustris* was first discovered from the rhizoplane of *Typha angustifolia* in the Danube river then isolated from pelagic waters[35, 36], while *K*. *palustris* had an optimal 2–7% NaCl requirement for growth[37]. Both strain GHF1031 (*Celeribacter baekdonensis*) and strain GHF1032 (*Albirhodobacter marinus*) are marine bacteria that belong to the family Rhodobacteraceae of the class Alphaproteobacteria, which was found to be one of the major phylogenetic assemblages of the ocean till date[38, 39]. Strain GHF2 (*Psychrobacter aquimaris*) and strains GHF10 and GHF1043 (*Psychrobacter cibarius*), all belonged to the genus *Psychrobacter* which were halotolerant bacteria and accommodated psychrophilic or psychrotolerant of growing environment[40, 41]. *Halomonas taeanensis*, (strain GHF11 and GHF1042) is a moderately halophilic bacterium isolated from a solar saltern in Korea[42]. Halophilic or halotolerant characteristics of these isolates can be expected to exploit novel bioflocculants which have a special adaptability to high salinity conditions[43–45].

EPS from representative strains were effective bioflocculants, which could be comparable to bioflocculant polysaccharides RPMP-1 and RPMP-2 in RPM [9]. EPS dosage-flocculation activity relation experiments showed that extracellular polysaccharides from strains GHF1, GHF1042, GHS20 and GHS21 exhibited FR of 51%, 49%, 53% and 55% respectively, while FR of RPMP-1 peaked at 25.4%, RPMP-2 exhibited a maximum FR of 35.5%, and FR of RPMP-1 and RPMP-2 combination showed a synergistic bioflocculation activity of 65.6%. EPS from representative strains may be the source of bioflocculant components in RPM, given their chemical composition similarity.

Composition analysis of polysaccharides from RPM and bacterial isolates provided convincing proof supporting that RPM flocculation components were of bacterial origin. Flocculation-active polysaccharides RPMP-1 and RPMP-2 from RPM were composed of Man, GlcN, Rha, GlcUA, GalUA, GalN, Glc, Gal, Xyl, Ara and Fuc[9], showing great similarity to that of bacterial isolate EPS. On the contrary, RPMP-1 and RPMP-2 exhibited totally different monosaccharide constitution from known polysaccharides purified from the *R*. *philippinarum* clam. Liu et al.[46] extracted polysaccharides from *R*. *philippinarum* and determined a composition of aminosugars, uronic acid, fucose and sulfate. Zhang et al.[47] isolated and characterized two water-soluble polysaccharides from *R*. *philippinarum* to be homoglucan–protein complexes. Additionally, RPMP-1 and RPMP-2 molecules showed no similarity with reported microbial bioflocculants for their complicated composition[9]. All these data referred the flocculation-active polysaccharides of RPM to an origin from associated bacteria rather than from other source within the *R*. *philippinarum* clam.

In summary, our results indicated that RPM from Zhoushan in China not only hosted a highly diverse bacterial community, but also held varied high-efficient bioflocculant-producing bacteria which could secret EPS with similar monosaccharide composition to RPM bioflocculation-active polysaccharides. Findings from Zhoushan RPM strongly supported that RPM flocculation components were of bacterial origin and make RPM reproduction possible by fermentation approach. Of note, the microbial community structure of RPM may be habitat-variable to some extent. A hypothesis is promoted that a "core microbiome" of functioning species exists in RPM samples from various habitats and looks forward to wide research[48–50].

## Supporting information

S1 Fig Rarefaction curve of the MiSeq 16S rDNA sequencing of the RPM samples. (TIF)

S1 Table The taxonomical classification and abundance of all OTUs from RPM. (DOCX)

S2 Table Phylogenetic identification of 14 bioflocculant-producing isolates based on 16S rDNA sequences and EzBioCloud’s database. (DOCX)

S3 Table Monosaccharide composition of EPS from bioflocculant-producing bacterial isolates. (DOCX)
