# Supplementary figures and images for: Microbial origin of bioflocculation components within a promising natural bioflocculant resource of Ruditapes philippinarum conglutination mud from an aquaculture farm in Zhoushan, China
Source: PLoS One. 2019 Jun 19;14(6):e0217679. doi: 10.1371/journal.pone.0217679 (PMC6583956; doi:10.1371/journal.pone.0217679)

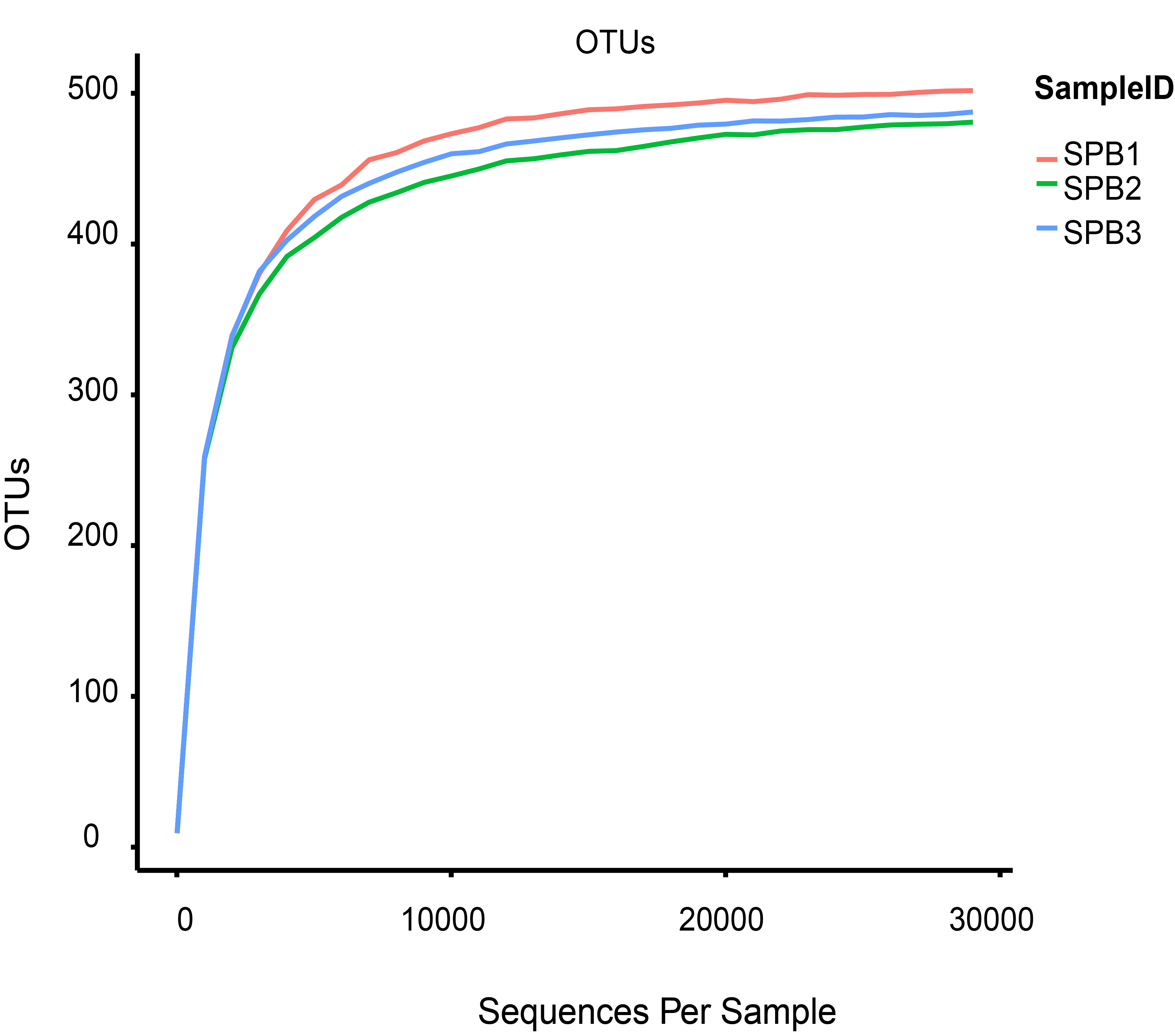

Supplement: S1 Fig — (TIF) [file pone.0217679.s001.tif]
