# Supplementary material for: Microbial origin of bioflocculation components within a promising natural bioflocculant resource of Ruditapes philippinarum conglutination mud from an aquaculture farm in Zhoushan, China
Source: PLoS One. 2019 Jun 19;14(6):e0217679. doi: 10.1371/journal.pone.0217679 (PMC6583956; doi:10.1371/journal.pone.0217679)
Supplement: S1 Table — (DOCX) [file pone.0217679.s002.docx]

**S1 Table. The taxonomical classification and abundance of all OTUs from RPM**

|  | RPM parallel samples | | |  |
| --- | --- | --- | --- | --- |
| OTU_ID | SPB1 | SPB2 | SPB3 | taxonomy |
| 1 | 1166 | 961 | 667 | k__Bacteria; p__Firmicutes; c__Clostridia; o__Clostridiales; f__Family_XII; g__Fusibacter |
| 2 | 52 | 65 | 19 | k__Bacteria; p__Proteobacteria; c__Alphaproteobacteria; o__Rhodobacterales; f__Rhodobacteraceae |
| 3 | 406 | 555 | 615 | k__Bacteria; p__Proteobacteria; c__Alphaproteobacteria; o__Rhizobiales; f__A0839; Ambiguous_taxa; Ambiguous_taxa |
| 4 | 2002 | 60 | 541 | k__Bacteria; p__Bacteroidetes; c__Bacteroidia; o__Bacteroidales; f__Porphyromonadaceae; g__uncultured; s__uncultured_Bacteroidetes_bacterium |
| 5 | 291 | 95 | 157 | k__Bacteria; p__Proteobacteria; c__Gammaproteobacteria; o__Vibrionales; f__Vibrionaceae; g__Vibrio |
| 6 | 23 | 23 | 18 | k__Bacteria; p__Cyanobacteria; c__Chloroplast; o__Euglena_agilis; f__Euglena_agilis; g__Euglena_agilis; s__Euglena_agilis |
| 7 | 34 | 40 | 50 | k__Bacteria; p__Proteobacteria; c__Gammaproteobacteria; o__KI89A_clade; f__uncultured_bacterium; g__uncultured_bacterium; s__uncultured_bacterium |
| 8 | 97 | 112 | 104 | k__Bacteria; p__Actinobacteria; c__Acidimicrobiia; o__Acidimicrobiales; f__Acidimicrobiaceae; g__CL500_29_marine_group; Ambiguous_taxa |
| 9 | 8 | 3 | 2 | k__Bacteria; p__Proteobacteria; c__Gammaproteobacteria; o__Oceanospirillales; f__SS1_B_06_26; g__uncultured_gamma_proteobacterium; s__uncultured_gamma_proteobacterium |
| 10 | 2810 | 2917 | 3292 | k__Bacteria; p__Proteobacteria; c__Alphaproteobacteria; o__SAR11_clade; f__LD12_freshwater_group; g__uncultured_bacterium; s__uncultured_bacterium |
| 11 | 46 | 56 | 44 | k__Bacteria; p__Proteobacteria; c__Gammaproteobacteria; o__Enterobacteriales; f__Enterobacteriaceae; g__Enterobacter |
| 12 | 28 | 12 | 19 | k__Bacteria; p__Bacteroidetes; c__Flavobacteriia; o__Flavobacteriales; f__Cryomorphaceae; g__Fluviicola |
| 13 | 1055 | 1202 | 1163 | k__Bacteria; p__Actinobacteria; c__Actinobacteria; o__Frankiales; f__Sporichthyaceae; g__hgcI_clade; s__uncultured_bacterium |
| 14 | 43 | 16 | 37 | k__Bacteria; p__Bacteroidetes; c__Bacteroidia; o__Bacteroidales; f__Marinilabiaceae; g__Marinifilum |
| 15 | 226 | 287 | 245 | k__Bacteria; p__Actinobacteria; c__Acidimicrobiia; o__Acidimicrobiales; f__Acidimicrobiaceae; g__CL500_29_marine_group; Ambiguous_taxa |
| 16 | 122 | 116 | 132 | k__Bacteria; p__Actinobacteria; c__Acidimicrobiia; o__Acidimicrobiales; f__Acidimicrobiaceae; g__CL500_29_marine_group; Ambiguous_taxa |
| 17 | 10 | 9 | 5 | k__Bacteria; p__Actinobacteria; c__Acidimicrobiia; o__Acidimicrobiales; f__uncultured |
| 18 | 374 | 35 | 155 | k__Bacteria; p__Proteobacteria; c__Gammaproteobacteria; o__Oceanospirillales; f__Oceanospirillaceae; g__Neptunomonas; Ambiguous_taxa |
| 19 | 99 | 4 | 24 | k__Bacteria; p__Firmicutes; c__Clostridia; o__Clostridiales; f__Family_XII; g__Fusibacter |
| 20 | 218 | 237 | 227 | k__Bacteria; p__Bacteroidetes; c__Sphingobacteriia; o__Sphingobacteriales; f__Saprospiraceae; g__Lewinella; Ambiguous_taxa |
| 21 | 86 | 92 | 85 | k__Bacteria; p__Proteobacteria; c__Alphaproteobacteria; o__Rhodobacterales; f__Rhodobacteraceae |
| 22 | 14 | 14 | 15 | k__Bacteria; p__Gemmatimonadetes; c__Gemmatimonadetes; o__Gemmatimonadales; f__Gemmatimonadaceae; g__Gemmatimonas; s__uncultured_bacterium |
| 23 | 163 | 5 | 30 | k__Bacteria; p__Firmicutes; c__Clostridia; o__Clostridiales; f__Family_XII; g__Fusibacter; s__uncultured_bacterium |
| 24 | 52 | 50 | 55 | k__Bacteria; p__Proteobacteria; c__Gammaproteobacteria; o__Aeromonadales; f__Aeromonadaceae; g__Aeromonas; Ambiguous_taxa |
| 25 | 122 | 234 | 88 | k__Bacteria; p__Bacteroidetes; c__Bacteroidia; o__Bacteroidales; f__Marinilabiaceae; g__Carboxylicivirga; s__uncultured_bacterium |
| 26 | 6 | 6 | 8 | k__Bacteria; p__Proteobacteria; c__Alphaproteobacteria; o__Kordiimonadales; f__Kordiimonadaceae; g__Kordiimonas; s__uncultured_bacterium |
| 27 | 6 | 4 | 6 | k__Bacteria; p__Bacteroidetes; c__Sphingobacteriia; o__Sphingobacteriales; f__CWT_CU03_E12; g__uncultured_bacterium; s__uncultured_bacterium |
| 28 | 50 | 57 | 56 | k__Bacteria; p__Actinobacteria; c__Acidimicrobiia; o__Acidimicrobiales; f__Acidimicrobiaceae; g__CL500_29_marine_group |
| 29 | 308 | 377 | 367 | k__Bacteria; p__Proteobacteria; c__Gammaproteobacteria; o__Xanthomonadales; f__Xanthomonadales_Incertae_Sedis; g__Acidibacter |
| 30 | 121 | 138 | 148 | k__Bacteria; p__Bacteroidetes; c__Sphingobacteriia; o__Sphingobacteriales; f__Saprospiraceae; g__Phaeodactylibacter; s__uncultured_bacterium |
| 31 | 527 | 656 | 587 | k__Bacteria; p__Chlorobi; c__Chlorobia; o__Chlorobiales; f__OPB56; g__uncultured_bacterium; s__uncultured_bacterium |
| 32 | 49 | 56 | 65 | k__Bacteria; p__Bacteroidetes; c__Sphingobacteriia; o__Sphingobacteriales; f__Saprospiraceae |
| 33 | 40 | 26 | 6 | k__Bacteria; p__Bacteroidetes; c__Flavobacteriia; o__Flavobacteriales; f__Flavobacteriaceae; g__uncultured; s__Bacteroidetes_bacterium_T4_KAD_str1 |
| 34 | 11 | 23 | 25 | k__Bacteria; p__Proteobacteria; c__Gammaproteobacteria; o__Alteromonadales; f__Shewanellaceae; g__Shewanella |
| 35 | 49 | 53 | 61 | k__Bacteria; p__Proteobacteria; c__Deltaproteobacteria |
| 36 | 69 | 80 | 87 | k__Bacteria; p__Proteobacteria; c__Betaproteobacteria; o__Methylophilales; f__Methylophilaceae; g__OM43_clade; s__uncultured_bacterium |
| 37 | 15 | 6 | 0 | k__Bacteria; p__Proteobacteria; c__Gammaproteobacteria; o__Alteromonadales; f__Alteromonadaceae |
| 38 | 5 | 12 | 8 | k__Bacteria; p__Proteobacteria; c__Alphaproteobacteria; o__Rickettsiales; f__Mitochondria; g__Nephroselmis_olivacea; s__Nephroselmis_olivacea |
| 39 | 105 | 19 | 17 | k__Bacteria; p__Proteobacteria; c__Epsilonproteobacteria; o__Campylobacterales; f__Campylobacteraceae; g__Arcobacter |
| 40 | 321 | 29 | 215 | k__Bacteria; p__Proteobacteria; c__Gammaproteobacteria; o__Vibrionales; f__Vibrionaceae; g__Vibrio; s__Vibrio_vulnificus |
| 41 | 29 | 15 | 15 | k__Bacteria; p__Firmicutes; c__Clostridia; o__Clostridiales |
| 42 | 36 | 0 | 2 | k__Bacteria; p__Fusobacteria; c__Fusobacteriia; o__Fusobacteriales; f__Fusobacteriaceae; g__Fusobacterium |
| 43 | 70 | 1 | 7 | k__Bacteria; p__Firmicutes; c__Erysipelotrichia; o__Erysipelotrichales; f__Erysipelotrichaceae |
| 44 | 916 | 114 | 245 | k__Bacteria; p__Proteobacteria; c__Epsilonproteobacteria; o__Campylobacterales; f__Campylobacteraceae; g__Arcobacter; Ambiguous_taxa |
| 45 | 73 | 5 | 36 | k__Bacteria; p__Firmicutes; c__Clostridia; o__Clostridiales; f__Family_XII; g__Fusibacter |
| 46 | 558 | 265 | 222 | k__Bacteria; p__Proteobacteria; c__Epsilonproteobacteria; o__Campylobacterales; f__Campylobacteraceae; g__Arcobacter; Ambiguous_taxa |
| 47 | 116 | 136 | 130 | k__Bacteria; p__Actinobacteria; c__Acidimicrobiia; o__Acidimicrobiales; f__Acidimicrobiaceae |
| 48 | 160 | 234 | 96 | k__Bacteria; p__Proteobacteria; c__Epsilonproteobacteria; o__Campylobacterales; f__Helicobacteraceae; g__Sulfurovum; s__uncultured_Sulfurovum_sp. |
| 49 | 330 | 300 | 46 | k__Bacteria; p__CKC4; Ambiguous_taxa; Ambiguous_taxa; Ambiguous_taxa; Ambiguous_taxa; Ambiguous_taxa |
| 50 | 16 | 25 | 18 | k__Bacteria; p__Proteobacteria; c__Deltaproteobacteria; o__Oligoflexales; f__Oligoflexaceae; g__uncultured_bacterium; s__uncultured_bacterium |
| 51 | 79 | 19 | 53 | k__Bacteria; p__Bacteroidetes; c__Flavobacteriia; o__Flavobacteriales; f__Flavobacteriaceae |
| 52 | 303 | 383 | 421 | k__Bacteria; p__Proteobacteria; c__Gammaproteobacteria; o__Vibrionales; f__Vibrionaceae; g__Vibrio |
| 53 | 240 | 274 | 252 | k__Bacteria; p__Bacteroidetes; c__Bacteroidetes_VC2.1_Bac22; Ambiguous_taxa; Ambiguous_taxa; Ambiguous_taxa; Ambiguous_taxa |
| 54 | 3 | 2 | 8 | k__Bacteria; p__Proteobacteria; c__Alphaproteobacteria; o__Rhizobiales; f__Bradyrhizobiaceae; g__Rhodopseudomonas |
| 55 | 455 | 566 | 489 | k__Bacteria; p__Bacteroidetes; c__Sphingobacteriia; o__Sphingobacteriales; f__Saprospiraceae; g__Phaeodactylibacter; s__uncultured_bacterium |
| 56 | 29 | 27 | 26 | k__Bacteria; p__Proteobacteria; c__SPOTSOCT00m83 |
| 57 | 195 | 19 | 10 | k__Bacteria; p__Firmicutes; c__Clostridia; o__Clostridiales; f__Family_XII; g__Fusibacter; s__uncultured_bacterium |
| 58 | 4 | 1 | 3 | k__Bacteria; p__Tenericutes; c__Mollicutes; o__NB1_n |
| 59 | 32 | 35 | 40 | k__Bacteria; p__Actinobacteria; c__Actinobacteria; o__Frankiales; f__Sporichthyaceae; g__hgcI_clade; s__uncultured_actinobacterium |
| 60 | 14 | 0 | 8 | k__Bacteria; p__Proteobacteria; c__Alphaproteobacteria; o__Rhodospirillales; f__Rhodospirillaceae; g__Candidatus_Riegeria; s__uncultured_bacterium |
| 61 | 17 | 0 | 2 | k__Bacteria; p__Bacteroidetes; c__Bacteroidia; o__Bacteroidales; f__Bacteroidaceae; g__Bacteroides |
| 62 | 661 | 780 | 775 | k__Bacteria; p__Proteobacteria; c__Betaproteobacteria; o__Burkholderiales; f__Alcaligenaceae; g__MWH_UniP1_aquatic_group |
| 63 | 37 | 48 | 54 | k__Bacteria; p__Actinobacteria; c__Thermoleophilia; o__Solirubrobacterales; f__480_2; g__uncultured_bacterium; s__uncultured_bacterium |
| 64 | 153 | 194 | 190 | k__Bacteria; p__Actinobacteria; c__Actinobacteria; o__PeM15; Ambiguous_taxa; Ambiguous_taxa; Ambiguous_taxa |
| 65 | 83 | 110 | 109 | k__Bacteria; p__Proteobacteria; c__Alphaproteobacteria; o__Rhodospirillales; f__Rhodospirillaceae; g__Roseospirillum; s__uncultured_bacterium |
| 66 | 11 | 29 | 17 | k__Bacteria; p__Bacteroidetes; c__Sphingobacteriia; o__Sphingobacteriales; f__Saprospiraceae; g__uncultured |
| 67 | 146 | 169 | 160 | k__Bacteria; p__Proteobacteria; c__Alphaproteobacteria; o__Rhodobacterales; f__Rhodobacteraceae; g__Tabrizicola; s__uncultured_bacterium |
| 68 | 50 | 37 | 39 | k__Bacteria; p__Bacteroidetes; c__Sphingobacteriia; o__Sphingobacteriales; f__NS11_12_marine_group; g__uncultured_bacterium; s__uncultured_bacterium |
| 69 | 90 | 109 | 114 | k__Bacteria; p__Proteobacteria; c__Betaproteobacteria; o__Burkholderiales; f__Burkholderiaceae; g__Lautropia; Ambiguous_taxa |
| 70 | 3 | 2 | 3 | k__Bacteria; p__Gemmatimonadetes; c__Gemmatimonadetes; o__Gemmatimonadales; f__Gemmatimonadaceae; g__uncultured |
| 71 | 87 | 128 | 148 | k__Bacteria; p__Bacteroidetes; c__Bacteroidia; o__Bacteroidales; f__Marinilabiaceae; g__Carboxylicivirga; s__uncultured_bacterium |
| 72 | 22 | 35 | 29 | k__Bacteria; p__Proteobacteria; c__Deltaproteobacteria; o__Bdellovibrionales; f__Bdellovibrionaceae; g__OM27_clade; s__uncultured_bacterium |
| 73 | 240 | 296 | 279 | k__Bacteria; p__Actinobacteria; c__Actinobacteria; o__Frankiales; f__Sporichthyaceae; g__hgcI_clade; s__uncultured_bacterium |
| 74 | 113 | 150 | 162 | k__Bacteria; p__Bacteroidetes; c__Cytophagia; o__Order_III; f__CK06_06_Mud_MAS4B_21; g__uncultured_bacterium; s__uncultured_bacterium |
| 75 | 209 | 266 | 281 | k__Bacteria; p__Proteobacteria; c__Betaproteobacteria; o__Burkholderiales; f__Comamonadaceae; g__Hydrogenophaga; Ambiguous_taxa |
| 76 | 687 | 759 | 785 | k__Bacteria; p__Actinobacteria; c__Actinobacteria; o__Micrococcales; f__Microbacteriaceae; g__Candidatus_Aquiluna; s__uncultured_bacterium |
| 77 | 151 | 191 | 170 | k__Bacteria; p__Actinobacteria; c__Actinobacteria; o__Frankiales; f__Sporichthyaceae; g__hgcI_clade; s__uncultured_bacterium |
| 78 | 26 | 17 | 23 | k__Bacteria; p__Bacteroidetes; c__Flavobacteriia; o__Flavobacteriales; f__NS9_marine_group; g__uncultured_bacterium; s__uncultured_bacterium |
| 79 | 41 | 25 | 47 | k__Bacteria; p__Chlorobi; c__Chlorobia; o__Chlorobiales; f__OPB56; g__uncultured_bacterium; s__uncultured_bacterium |
| 80 | 390 | 428 | 472 | k__Bacteria; p__Actinobacteria; c__Acidimicrobiia; o__Acidimicrobiales; f__Acidimicrobiaceae; g__CL500_29_marine_group |
| 81 | 8 | 3 | 3 | k__Bacteria; p__Bacteroidetes; c__Flavobacteriia; o__Flavobacteriales; f__Cryomorphaceae; g__Owenweeksia |
| 82 | 18 | 8 | 10 | k__Bacteria; p__Proteobacteria; c__Alphaproteobacteria; o__Rhodobacterales; f__Rhodobacteraceae |
| 83 | 145 | 180 | 174 | k__Bacteria; p__Bacteroidetes; c__Sphingobacteriia; o__Sphingobacteriales; f__Saprospiraceae; g__uncultured; Ambiguous_taxa |
| 84 | 255 | 237 | 281 | k__Bacteria; p__Chlorobi; c__Chlorobia; o__Chlorobiales; f__OPB56 |
| 85 | 151 | 293 | 350 | k__Bacteria; p__Firmicutes; c__Bacilli; o__Bacillales; f__Bacillaceae; g__Bacillus; s__Bacillus_cereus |
| 86 | 51 | 4 | 9 | k__Bacteria; p__Firmicutes; c__Clostridia; o__Clostridiales; f__Family_XII; g__Fusibacter; s__bioreactor_metagenome |
| 87 | 77 | 158 | 193 | k__Bacteria; p__Firmicutes; c__Bacilli; o__Lactobacillales; f__Streptococcaceae; g__Lactococcus; s__uncultured_bacterium |
| 88 | 12 | 0 | 2 | k__Bacteria; p__Tenericutes; c__Mollicutes |
| 89 | 108 | 13 | 12 | k__Bacteria; p__Bacteroidetes; c__Flavobacteriia; o__Flavobacteriales; f__Flavobacteriaceae; g__Mesoflavibacter; Ambiguous_taxa |
| 90 | 29 | 39 | 37 | k__Bacteria; p__Bacteroidetes; c__Cytophagia; o__Cytophagales; f__Cyclobacteriaceae; g__uncultured; Ambiguous_taxa |
| 91 | 89 | 131 | 111 | k__Bacteria; p__Bacteroidetes |
| 92 | 38 | 4 | 20 | k__Bacteria; p__Proteobacteria; c__Gammaproteobacteria; o__Oceanospirillales; f__Oceanospirillaceae |
| 93 | 6 | 4 | 7 | k__Bacteria; p__Proteobacteria; c__Gammaproteobacteria; o__Thiotrichales; f__Thiotrichales_Incertae_Sedis; g__Caedibacter; s__Caedibacter_taeniospiralis |
| 94 | 108 | 120 | 161 | k__Bacteria; p__Proteobacteria; c__Alphaproteobacteria; o__Rhizobiales; f__MNG7 |
| 95 | 28 | 35 | 34 | k__Bacteria; p__Bacteroidetes; c__Sphingobacteriia; o__Sphingobacteriales; f__Saprospiraceae; g__uncultured |
| 96 | 6 | 10 | 11 | k__Bacteria; p__Bacteroidetes; c__Sphingobacteriia; o__Sphingobacteriales; f__Chitinophagaceae; g__uncultured |
| 97 | 251 | 277 | 263 | k__Bacteria; p__Proteobacteria; c__Alphaproteobacteria; o__Rhodospirillales; f__Acetobacteraceae; g__Roseomonas; s__uncultured_bacterium |
| 98 | 23 | 27 | 23 | k__Bacteria; p__Bacteroidetes; c__Cytophagia; o__Cytophagales; f__Cytophagaceae; g__uncultured |
| 99 | 11 | 12 | 30 | k__Bacteria; p__Bacteroidetes; c__Flavobacteriia; o__Flavobacteriales; f__Flavobacteriaceae; g__Polaribacter; s__Polaribacter_gangjinensis |
| 100 | 189 | 9 | 4 | k__Bacteria; p__Bacteroidetes; c__Flavobacteriia; o__Flavobacteriales; f__Flavobacteriaceae; g__Flavobacterium; s__Flavobacterium_dongtanense |
| 101 | 36 | 17 | 40 | k__Bacteria; p__Bacteroidetes; c__Bacteroidia; o__Bacteroidales; f__Marinilabiaceae; g__Marinifilum; Ambiguous_taxa |
| 102 | 3 | 3 | 4 | k__Bacteria; p__Proteobacteria; c__Alphaproteobacteria; o__Rhodobacterales; f__Rhodobacteraceae |
| 103 | 7 | 8 | 5 | k__Bacteria; p__Acidobacteria; c__Holophagae; o__Subgroup_10; f__ABS_19; g__uncultured_bacterium; s__uncultured_bacterium |
| 104 | 55 | 51 | 34 | k__Bacteria; p__Bacteroidetes; c__Flavobacteriia; o__Flavobacteriales; f__Flavobacteriaceae; g__Actibacter; s__uncultured_Bacteroidetes_bacterium |
| 105 | 30 | 8 | 16 | k__Bacteria; p__Bacteroidetes; c__Flavobacteriia; o__Flavobacteriales; f__Flavobacteriaceae; g__Flavobacterium |
| 106 | 151 | 198 | 215 | k__Bacteria; p__Bacteroidetes; c__Sphingobacteriia; o__Sphingobacteriales; f__Saprospiraceae |
| 107 | 225 | 272 | 260 | k__Bacteria; p__Actinobacteria; c__Acidimicrobiia; o__Acidimicrobiales; f__Acidimicrobiaceae; g__CL500_29_marine_group; s__uncultured_bacterium |
| 108 | 15 | 23 | 24 | k__Bacteria; p__Bacteroidetes; c__Flavobacteriia; o__Flavobacteriales; f__Cryomorphaceae; g__NS10_marine_group; s__uncultured_bacterium |
| 109 | 41 | 27 | 31 | k__Bacteria; p__Actinobacteria; c__Actinobacteria; o__Micrococcales; f__Microbacteriaceae; g__Alpinimonas; s__uncultured_bacterium |
| 110 | 120 | 142 | 146 | k__Bacteria; p__Gemmatimonadetes; c__Gemmatimonadetes; o__Gemmatimonadales; f__Gemmatimonadaceae; g__Gemmatimonas |
| 111 | 7 | 18 | 14 | k__Bacteria; p__Proteobacteria; c__Alphaproteobacteria; o__Rickettsiales; f__SM2D12; g__uncultured_bacterium; s__uncultured_bacterium |
| 112 | 7 | 6 | 1 | k__Bacteria; p__Proteobacteria; c__Alphaproteobacteria; o__Caulobacterales; f__Hyphomonadaceae; g__Woodsholea; s__uncultured_bacterium |
| 113 | 30 | 30 | 44 | k__Bacteria; p__Proteobacteria; c__Deltaproteobacteria; o__Desulfuromonadales; f__GR_WP33_58 |
| 114 | 214 | 180 | 214 | k__Bacteria; p__Actinobacteria; c__Actinobacteria; o__PeM15; Ambiguous_taxa; Ambiguous_taxa; Ambiguous_taxa |
| 115 | 46 | 58 | 60 | k__Bacteria; p__Bacteroidetes; c__Sphingobacteriia; o__Sphingobacteriales; f__Chitinophagaceae; g__Sediminibacterium; Ambiguous_taxa |
| 116 | 80 | 104 | 112 | k__Bacteria; p__Bacteroidetes; c__Sphingobacteriia; o__Sphingobacteriales; f__Saprospiraceae; g__uncultured; s__uncultured_Sphingobacteriales_bacterium |
| 117 | 180 | 221 | 219 | k__Bacteria; p__Chlorobi; c__Chlorobia; o__Chlorobiales; f__OPB56; g__uncultured_bacterium; s__uncultured_bacterium |
| 118 | 47 | 55 | 54 | k__Bacteria; p__Bacteroidetes; c__Sphingobacteriia; o__Sphingobacteriales; f__Chitinophagaceae; g__uncultured; Ambiguous_taxa |
| 119 | 44 | 55 | 63 | k__Bacteria; p__Proteobacteria; c__Betaproteobacteria; o__Burkholderiales; f__Alcaligenaceae; g__GKS98_freshwater_group; s__uncultured_bacterium |
| 120 | 8 | 24 | 25 | k__Bacteria; p__Proteobacteria; c__Deltaproteobacteria; o__Bdellovibrionales; f__Bdellovibrionaceae; g__Bdellovibrio; s__uncultured_bacterium |
| 121 | 25 | 14 | 5 | k__Bacteria; p__Proteobacteria; c__Gammaproteobacteria; o__Order_Incertae_Sedis; f__Family_Incertae_Sedis; g__Marinicella; s__uncultured_bacterium |
| 122 | 62 | 96 | 62 | k__Bacteria; p__Proteobacteria; c__Deltaproteobacteria; o__Desulfuromonadales; f__GR_WP33_58; g__uncultured_bacterium_GR_WP33_58; s__uncultured_bacterium_GR_WP33_58 |
| 123 | 2 | 2 | 3 | k__Bacteria; p__Cyanobacteria; c__Chloroplast |
| 124 | 59 | 69 | 75 | k__Bacteria; p__Proteobacteria; c__Deltaproteobacteria; o__Myxococcales; f__Sandaracinaceae; g__uncultured; s__uncultured_bacterium |
| 125 | 15 | 13 | 20 | k__Bacteria; p__Proteobacteria; c__Alphaproteobacteria; o__Rhizobiales; f__Hyphomicrobiaceae; g__Pedomicrobium; s__uncultured_bacterium |
| 126 | 82 | 50 | 27 | k__Bacteria; p__Proteobacteria; c__Gammaproteobacteria; o__Alteromonadales; f__Alteromonadaceae; g__Alteromonas |
| 127 | 41 | 45 | 37 | k__Bacteria; p__Proteobacteria; c__Deltaproteobacteria; o__Bdellovibrionales; f__Bdellovibrionaceae; g__OM27_clade; s__uncultured_bacterium |
| 128 | 80 | 98 | 81 | k__Bacteria; p__Acidobacteria; c__Acidobacteria; o__Subgroup_3; f__SJA_149; g__uncultured_bacterium; s__uncultured_bacterium |
| 129 | 12 | 17 | 14 | k__Bacteria; p__Cyanobacteria; c__Chloroplast |
| 130 | 504 | 607 | 567 | k__Bacteria; p__Proteobacteria; c__Betaproteobacteria; o__Burkholderiales; f__Comamonadaceae |
| 131 | 58 | 49 | 55 | k__Bacteria; p__Cyanobacteria; c__Chloroplast |
| 132 | 14 | 0 | 4 | k__Bacteria; p__Firmicutes; c__Clostridia; o__Clostridiales; f__Family_XII; g__Fusibacter; s__uncultured_bacterium |
| 133 | 17 | 20 | 15 | k__Bacteria; p__Actinobacteria; c__Actinobacteria; o__Frankiales; f__Sporichthyaceae; g__hgcI_clade |
| 134 | 18 | 1 | 17 | k__Bacteria; p__Bacteroidetes; c__Flavobacteriia; o__Flavobacteriales; f__Flavobacteriaceae |
| 135 | 100 | 83 | 85 | k__Bacteria; p__Proteobacteria; c__Alphaproteobacteria; o__Rhodobacterales; f__Rhodobacteraceae |
| 136 | 20 | 18 | 22 | k__Bacteria; p__Proteobacteria; c__Alphaproteobacteria; o__Rhodospirillales; f__Acetobacteraceae; g__Roseomonas; s__uncultured_bacterium |
| 137 | 22 | 17 | 29 | k__Bacteria; p__Proteobacteria; c__Deltaproteobacteria; o__Myxococcales; f__Blfdi19 |
| 138 | 7 | 10 | 7 | k__Bacteria; p__Cyanobacteria; c__ML635J_21; o__uncultured_bacterium; f__uncultured_bacterium; g__uncultured_bacterium; s__uncultured_bacterium |
| 139 | 139 | 165 | 147 | k__Bacteria; p__Proteobacteria; c__Betaproteobacteria; o__Burkholderiales; f__Comamonadaceae |
| 140 | 45 | 57 | 43 | k__Bacteria; p__Proteobacteria; c__Betaproteobacteria; o__Burkholderiales; f__Comamonadaceae |
| 141 | 7 | 15 | 16 | k__Bacteria; p__Proteobacteria; c__Deltaproteobacteria; o__Bdellovibrionales; f__Bacteriovoracaceae; g__Peredibacter; s__uncultured_bacterium |
| 142 | 35 | 32 | 32 | k__Bacteria; p__Bacteroidetes; c__Sphingobacteriia; o__Sphingobacteriales; f__Chitinophagaceae; g__uncultured |
| 143 | 14 | 22 | 25 | k__Bacteria; p__Actinobacteria; c__Nitriliruptoria; o__Nitriliruptorales; f__Nitriliruptoraceae; g__Nitriliruptor |
| 144 | 9 | 4 | 2 | k__Bacteria; p__Bacteroidetes; c__Cytophagia; o__Cytophagales; f__Flammeovirgaceae; g__Fabibacter; Ambiguous_taxa |
| 145 | 84 | 77 | 106 | k__Bacteria; p__Proteobacteria; c__Betaproteobacteria; o__Methylophilales; f__Methylophilaceae; g__LD28_freshwater_group |
| 146 | 132 | 173 | 169 | k__Bacteria; p__Bacteroidetes; c__Cytophagia; o__Order_III; f__CK06_06_Mud_MAS4B_21 |
| 147 | 12 | 8 | 15 | k__Bacteria; p__Proteobacteria; c__Deltaproteobacteria; o__Myxococcales; f__Cystobacteraceae; g__Anaeromyxobacter; s__uncultured_bacterium |
| 148 | 12 | 3 | 3 | k__Bacteria; p__Firmicutes; c__Clostridia; o__Clostridiales |
| 149 | 18 | 35 | 26 | k__Bacteria; p__Proteobacteria; c__Betaproteobacteria; o__Methylophilales; f__Methylophilaceae |
| 150 | 261 | 255 | 237 | k__Bacteria; p__Bacteroidetes; c__Cytophagia; o__Cytophagales; f__Cyclobacteriaceae |
| 151 | 106 | 55 | 39 | k__Bacteria; p__Bacteroidetes; c__Flavobacteriia; o__Flavobacteriales; f__Flavobacteriaceae; g__uncultured |
| 152 | 29 | 11 | 11 | k__Bacteria; p__Actinobacteria; c__Acidimicrobiia; o__Acidimicrobiales; f__Acidimicrobiaceae; g__Illumatobacter; Ambiguous_taxa |
| 153 | 76 | 67 | 54 | k__Bacteria; p__Proteobacteria; c__Alphaproteobacteria; o__Rhizobiales |
| 154 | 90 | 146 | 132 | k__Bacteria; p__Proteobacteria; c__Alphaproteobacteria; o__Rhodospirillales; f__I_10; g__uncultured_bacterium; s__uncultured_bacterium |
| 155 | 1 | 2 | 0 | k__Bacteria; p__Bacteroidetes; c__Sphingobacteriia; o__Sphingobacteriales; f__Saprospiraceae |
| 156 | 24 | 7 | 21 | k__Bacteria; p__Bacteroidetes; c__Sphingobacteriia; o__Sphingobacteriales; f__NS11_12_marine_group; g__uncultured_bacterium; s__uncultured_bacterium |
| 157 | 68 | 8 | 8 | k__Bacteria; p__Proteobacteria; c__Gammaproteobacteria; o__Oceanospirillales; f__Oceanospirillaceae |
| 158 | 31 | 34 | 28 | k__Bacteria; p__Cyanobacteria; c__Chloroplast |
| 159 | 14 | 19 | 21 | k__Bacteria; p__Proteobacteria; c__TA18 |
| 160 | 28 | 24 | 20 | k__Bacteria; p__Bacteroidetes; c__Cytophagia; o__Order_III; f__BIgi5; g__uncultured_bacterium; s__uncultured_bacterium |
| 161 | 42 | 77 | 51 | k__Bacteria; p__Cyanobacteria; c__Cyanobacteria; o__SubsectionI; f__FamilyI |
| 162 | 41 | 47 | 56 | k__Bacteria; p__Proteobacteria; c__TA18; o__uncultured_bacterium; f__uncultured_bacterium; g__uncultured_bacterium; s__uncultured_bacterium |
| 163 | 33 | 6 | 1 | k__Bacteria; p__Bacteroidetes; c__Bacteroidia; o__Bacteroidales; f__Marinilabiaceae; g__Marinifilum; s__uncultured_bacterium |
| 164 | 98 | 123 | 97 | k__Bacteria; p__Proteobacteria; c__Betaproteobacteria; o__Burkholderiales; f__Comamonadaceae |
| 165 | 15 | 25 | 18 | k__Bacteria; p__Proteobacteria; c__Gammaproteobacteria; o__Alteromonadales; f__Pseudoalteromonadaceae; g__Pseudoalteromonas; Ambiguous_taxa |
| 166 | 34 | 68 | 58 | k__Bacteria; p__Proteobacteria; c__Deltaproteobacteria; o__GR_WP33_30 |
| 167 | 1 | 2 | 0 | k__Bacteria; p__Proteobacteria; c__Alphaproteobacteria; o__SAR11_clade; Ambiguous_taxa; Ambiguous_taxa; Ambiguous_taxa |
| 168 | 31 | 21 | 22 | k__Bacteria; p__Actinobacteria; c__Acidimicrobiia; o__Acidimicrobiales; f__Acidimicrobiaceae |
| 169 | 37 | 24 | 33 | k__Bacteria; p__Proteobacteria; c__Alphaproteobacteria; o__Rhodobacterales; f__Rhodobacteraceae |
| 170 | 27 | 3 | 12 | k__Bacteria; p__Proteobacteria; c__Deltaproteobacteria; o__Desulfobacterales; f__Desulfobulbaceae |
| 171 | 68 | 1 | 48 | k__Bacteria; p__Bacteroidetes; c__Bacteroidia; o__Bacteroidia_Incertae_Sedis; f__Prolixibacteraceae; g__Prolixibacter; Ambiguous_taxa |
| 172 | 38 | 22 | 33 | k__Bacteria; p__Bacteroidetes; c__Sphingobacteriia; o__Sphingobacteriales; f__Chitinophagaceae; g__uncultured; s__uncultured_bacterium |
| 173 | 11 | 9 | 3 | k__Bacteria; p__Proteobacteria; c__Alphaproteobacteria; o__Rhodobacterales; f__Rhodobacteraceae |
| 174 | 21 | 19 | 22 | k__Bacteria; p__Bacteroidetes; c__Cytophagia; o__Order_III; f__CK06_06_Mud_MAS4B_21; g__uncultured_bacterium; s__uncultured_bacterium |
| 175 | 123 | 162 | 149 | k__Bacteria; p__Bacteroidetes; c__Flavobacteriia; o__Flavobacteriales; f__NS9_marine_group; Ambiguous_taxa; Ambiguous_taxa |
| 176 | 16 | 15 | 18 | k__Bacteria; p__Proteobacteria; c__Alphaproteobacteria; o__Rhodospirillales; f__Rhodospirillaceae; g__Insolitispirillum |
| 177 | 70 | 78 | 80 | k__Bacteria; p__Gemmatimonadetes; c__Gemmatimonadetes; o__Gemmatimonadales; f__Gemmatimonadaceae |
| 178 | 53 | 53 | 82 | k__Bacteria; p__Firmicutes; c__Clostridia; o__Clostridiales; f__Family_XII; g__Fusibacter; s__uncultured_bacterium |
| 179 | 6 | 7 | 4 | k__Bacteria; p__Proteobacteria; c__Alphaproteobacteria; o__Sphingomonadales; f__Sphingomonadaceae; g__Sphingomonas; s__unidentified_marine_bacterioplankton |
| 180 | 119 | 125 | 122 | k__Bacteria; p__Proteobacteria; c__Alphaproteobacteria; o__Rhodobacterales; f__Rhodobacteraceae |
| 181 | 13 | 0 | 1 | k__Bacteria; p__Firmicutes; c__Clostridia; o__Clostridiales; f__Peptostreptococcaceae; g__Proteocatella; s__uncultured_bacterium |
| 182 | 4 | 6 | 0 | k__Bacteria; p__Bacteroidetes; c__Flavobacteriia; o__Flavobacteriales; f__Cryomorphaceae; g__Fluviicola |
| 183 | 3 | 0 | 1 | k__Bacteria; p__Firmicutes; c__Clostridia; o__Clostridiales; f__Lachnospiraceae |
| 184 | 6 | 5 | 7 | k__Bacteria; p__Proteobacteria; c__Betaproteobacteria; o__Hydrogenophilales; f__Hydrogenophilaceae; g__uncultured; s__uncultured_beta_proteobacterium |
| 185 | 40 | 50 | 42 | k__Bacteria; p__Proteobacteria; c__Deltaproteobacteria; o__Desulfuromonadales |
| 186 | 19 | 40 | 32 | k__Bacteria; p__Proteobacteria; c__Deltaproteobacteria; o__Desulfuromonadales; f__GR_WP33_58 |
| 187 | 95 | 108 | 119 | k__Bacteria; p__Proteobacteria; c__Betaproteobacteria; o__Burkholderiales; f__Comamonadaceae |
| 188 | 42 | 7 | 5 | k__Bacteria; p__Bacteroidetes |
| 189 | 104 | 151 | 176 | k__Bacteria; p__Proteobacteria; c__Betaproteobacteria; o__Nitrosomonadales; f__Nitrosomonadaceae; g__uncultured |
| 190 | 12 | 15 | 21 | k__Bacteria; p__Firmicutes; c__Bacilli; o__Lactobacillales; f__Enterococcaceae; g__Enterococcus; Ambiguous_taxa |
| 191 | 130 | 144 | 172 | k__Bacteria; p__Proteobacteria; c__Alphaproteobacteria; o__Caulobacterales; f__Hyphomonadaceae; g__Hyphomonas; Ambiguous_taxa |
| 192 | 16 | 0 | 7 | k__Bacteria; p__Bacteroidetes; c__Bacteroidetes_VC2.1_Bac22 |
| 193 | 35 | 47 | 39 | k__Bacteria; p__Proteobacteria; c__Gammaproteobacteria; o__Pseudomonadales; f__Pseudomonadaceae; g__Pseudomonas |
| 194 | 3 | 1 | 3 | k__Bacteria; p__Proteobacteria; c__Deltaproteobacteria |
| 195 | 98 | 110 | 117 | k__Bacteria; p__Actinobacteria; c__Actinobacteria; o__Frankiales; f__Sporichthyaceae; g__hgcI_clade |
| 196 | 51 | 81 | 88 | k__Bacteria; p__Proteobacteria; c__Alphaproteobacteria; o__Rickettsiales; f__Rickettsiaceae; g__uncultured; Ambiguous_taxa |
| 197 | 17 | 24 | 34 | k__Bacteria; p__Bacteroidetes; c__Flavobacteriia; o__Flavobacteriales; f__NS9_marine_group; g__uncultured_bacterium; s__uncultured_bacterium |
| 198 | 60 | 49 | 56 | k__Bacteria; p__Cyanobacteria; c__Cyanobacteria; o__SubsectionI; f__FamilyI; g__Synechococcus; Ambiguous_taxa |
| 199 | 106 | 115 | 132 | k__Bacteria; p__Bacteroidetes; c__Sphingobacteriia; o__Sphingobacteriales; f__NS11_12_marine_group; g__uncultured_Sphingobacterium_sp.; s__uncultured_Sphingobacterium_sp. |
| 200 | 132 | 113 | 106 | k__Bacteria; p__Actinobacteria; c__Acidimicrobiia; o__Acidimicrobiales; f__Acidimicrobiaceae; g__CL500_29_marine_group; Ambiguous_taxa |
| 201 | 19 | 17 | 17 | k__Bacteria; p__Cyanobacteria; c__Chloroplast; o__Porphyridium_purpureum; f__Porphyridium_purpureum; g__Porphyridium_purpureum; s__Porphyridium_purpureum |
| 202 | 28 | 45 | 41 | k__Bacteria; p__Bacteroidetes; c__Flavobacteriia; o__Flavobacteriales; f__Flavobacteriaceae; g__Flavobacterium |
| 203 | 33 | 29 | 41 | k__Bacteria; p__Proteobacteria; c__Gammaproteobacteria; o__Pseudomonadales; f__Moraxellaceae; g__Acinetobacter |
| 204 | 14 | 28 | 27 | k__Bacteria; p__Actinobacteria; c__Acidimicrobiia; o__Acidimicrobiales; f__Sva0996_marine_group; Ambiguous_taxa; Ambiguous_taxa |
| 205 | 79 | 16 | 15 | k__Bacteria; p__Bacteroidetes; c__Bacteroidia; o__Bacteroidia_Incertae_Sedis; f__Draconibacteriaceae; g__Draconibacterium; s__uncultured_bacterium |
| 206 | 28 | 37 | 35 | k__Bacteria; p__Actinobacteria; c__Actinobacteria; o__Frankiales; f__Sporichthyaceae; g__hgcI_clade |
| 207 | 18 | 21 | 13 | k__Bacteria; p__Proteobacteria; c__Alphaproteobacteria; o__Caulobacterales; f__Hyphomonadaceae; g__Hirschia; s__uncultured_bacterium |
| 208 | 20 | 0 | 2 | k__Bacteria; p__Firmicutes; c__Clostridia; o__Clostridiales; f__Lachnospiraceae |
| 209 | 7 | 11 | 9 | k__Bacteria; p__Actinobacteria; c__Actinobacteria; o__Bifidobacteriales; f__Bifidobacteriaceae; g__Bifidobacterium; Ambiguous_taxa |
| 210 | 176 | 224 | 225 | k__Bacteria; p__Chlorobi; c__Chlorobia; o__Chlorobiales; f__OPB56; g__uncultured_bacterium; s__uncultured_bacterium |
| 211 | 46 | 56 | 40 | k__Bacteria; p__Proteobacteria; c__Deltaproteobacteria; o__Bdellovibrionales; f__Bdellovibrionaceae; g__OM27_clade; s__uncultured_bacterium |
| 212 | 98 | 115 | 100 | k__Bacteria; p__Bacteroidetes; c__Flavobacteriia; o__Flavobacteriales; f__Cryomorphaceae; g__Owenweeksia; s__uncultured_bacterium |
| 213 | 124 | 157 | 175 | k__Bacteria; p__Bacteroidetes; c__Sphingobacteriia; o__Sphingobacteriales; f__Saprospiraceae; g__Haliscomenobacter; s__uncultured_bacterium |
| 214 | 35 | 49 | 55 | k__Bacteria; p__Bacteroidetes; c__Sphingobacteriia; o__Sphingobacteriales; f__env.OPS_17 |
| 215 | 29 | 42 | 43 | k__Bacteria; p__Actinobacteria; c__Acidimicrobiia; o__Acidimicrobiales; f__uncultured |
| 216 | 22 | 28 | 20 | k__Bacteria; p__Bacteroidetes; c__Sphingobacteriia; o__Sphingobacteriales; f__Chitinophagaceae |
| 217 | 11 | 7 | 24 | k__Bacteria; p__Actinobacteria; c__Acidimicrobiia; o__Acidimicrobiales; f__Acidimicrobiaceae; g__CL500_29_marine_group |
| 218 | 10 | 17 | 12 | k__Bacteria; p__Proteobacteria; c__Alphaproteobacteria; o__Rickettsiales; f__Rickettsiaceae |
| 219 | 9 | 19 | 11 | k__Bacteria; p__Proteobacteria; c__Deltaproteobacteria |
| 220 | 27 | 28 | 40 | k__Bacteria; p__Proteobacteria; c__Gammaproteobacteria; o__Enterobacteriales; f__Enterobacteriaceae; g__Morganella; s__uncultured_bacterium |
| 221 | 14 | 16 | 23 | k__Bacteria; p__Proteobacteria; c__Alphaproteobacteria; o__Rhodospirillales; f__Rhodospirillales_Incertae_Sedis; g__Elioraea |
| 222 | 11 | 20 | 12 | k__Bacteria; p__Cyanobacteria; c__Chloroplast |
| 223 | 31 | 0 | 0 | k__Bacteria; p__Proteobacteria; c__Alphaproteobacteria; o__Rhodospirillales; f__Rhodospirillaceae; g__Thalassospira; s__uncultured_Epulopiscium_sp. |
| 224 | 19 | 26 | 31 | k__Bacteria; p__Cyanobacteria; c__Chloroplast |
| 225 | 53 | 16 | 8 | k__Bacteria; p__Proteobacteria; c__Gammaproteobacteria; o__Alteromonadales; f__Shewanellaceae; g__Shewanella |
| 226 | 36 | 40 | 38 | k__Bacteria; p__Proteobacteria; c__Deltaproteobacteria |
| 227 | 12 | 12 | 13 | k__Bacteria; p__Proteobacteria; c__Alphaproteobacteria; o__Caulobacterales; f__Hyphomonadaceae |
| 228 | 41 | 40 | 42 | k__Bacteria; p__Actinobacteria; c__Actinobacteria; o__Frankiales; f__Sporichthyaceae; g__hgcI_clade; Ambiguous_taxa |
| 229 | 17 | 21 | 10 | k__Bacteria; p__Gracilibacteria; c__uncultured_epsilon_proteobacterium; o__uncultured_epsilon_proteobacterium; f__uncultured_epsilon_proteobacterium; g__uncultured_epsilon_proteobacterium; s__uncultured_epsilon_proteobacterium |
| 230 | 67 | 67 | 80 | k__Bacteria; p__Bacteroidetes; c__Flavobacteriia; o__Flavobacteriales; f__Cryomorphaceae; g__Fluviicola; s__uncultured_Flexibacter_sp. |
| 231 | 21 | 2 | 31 | k__Bacteria; p__Proteobacteria; c__Gammaproteobacteria; o__Oceanospirillales; f__Oceanospirillaceae; g__Marinobacterium; s__uncultured_Marinobacterium_sp. |
| 232 | 53 | 35 | 13 | k__Bacteria; p__Proteobacteria; c__Alphaproteobacteria; o__Rhodobacterales; f__Rhodobacteraceae |
| 233 | 61 | 54 | 52 | k__Bacteria; p__Proteobacteria; c__Gammaproteobacteria; o__34P16; f__gamma_proteobacterium_HdN1; g__gamma_proteobacterium_HdN1; s__gamma_proteobacterium_HdN1 |
| 234 | 55 | 103 | 81 | k__Bacteria; p__Proteobacteria; c__Betaproteobacteria; o__Burkholderiales; f__Comamonadaceae |
| 235 | 38 | 33 | 39 | k__Bacteria; p__Proteobacteria; c__Betaproteobacteria; o__Methylophilales; f__Methylophilaceae |
| 236 | 55 | 85 | 90 | k__Bacteria; p__Proteobacteria; c__Betaproteobacteria; o__Burkholderiales; f__Comamonadaceae |
| 237 | 35 | 51 | 40 | k__Bacteria; p__Actinobacteria; c__Acidimicrobiia; o__Acidimicrobiales; f__uncultured; Ambiguous_taxa; Ambiguous_taxa |
| 238 | 53 | 77 | 73 | k__Bacteria; p__Proteobacteria; c__Betaproteobacteria; o__Burkholderiales; f__Burkholderiaceae; g__Polynucleobacter; Ambiguous_taxa |
| 239 | 103 | 142 | 181 | k__Bacteria; p__Proteobacteria; c__TA18 |
| 240 | 26 | 15 | 22 | k__Bacteria; p__Bacteroidetes; c__Flavobacteriia; o__Flavobacteriales; f__Cryomorphaceae; g__Fluviicola; s__uncultured_bacterium |
| 241 | 66 | 64 | 87 | k__Bacteria; p__Proteobacteria; c__Deltaproteobacteria; o__Desulfuromonadales; f__GR_WP33_58; g__uncultured_bacterium; s__uncultured_bacterium |
| 242 | 26 | 26 | 39 | k__Bacteria; p__Bacteroidetes; c__Flavobacteriia; o__Flavobacteriales; f__Cryomorphaceae; g__Owenweeksia; Ambiguous_taxa |
| 243 | 10 | 3 | 7 | k__Bacteria; p__Bacteroidetes |
| 244 | 6 | 5 | 8 | k__Bacteria; p__Bacteroidetes; c__Sphingobacteriia; o__Sphingobacteriales; f__Sphingobacteriaceae; g__Solitalea; s__uncultured_bacterium |
| 245 | 3 | 0 | 3 | k__Bacteria; p__Firmicutes; c__Clostridia; o__Clostridiales; f__Ruminococcaceae; g__Subdoligranulum; s__uncultured_bacterium |
| 246 | 24 | 41 | 20 | k__Bacteria; p__Bacteroidetes |
| 247 | 70 | 96 | 89 | k__Bacteria; p__Bacteroidetes; c__Sphingobacteriia; o__Sphingobacteriales; f__Saprospiraceae; g__uncultured |
| 248 | 30 | 35 | 30 | k__Bacteria; p__Proteobacteria; c__Alphaproteobacteria; o__Caulobacterales; f__Hyphomonadaceae; g__Woodsholea; s__uncultured_bacterium |
| 249 | 34 | 0 | 5 | k__Bacteria; p__Proteobacteria; c__Gammaproteobacteria; o__Oceanospirillales; f__Oceanospirillaceae; g__Neptuniibacter; Ambiguous_taxa |
| 250 | 36 | 16 | 14 | k__Bacteria; p__Bacteroidetes; c__Flavobacteriia; o__Flavobacteriales; f__Flavobacteriaceae |
| 251 | 12 | 12 | 8 | k__Bacteria; p__Bacteroidetes; c__Sphingobacteriia; o__Sphingobacteriales; f__LiUU_11_161; g__uncultured_bacterium; s__uncultured_bacterium |
| 252 | 9 | 10 | 12 | k__Bacteria; p__Actinobacteria; c__Nitriliruptoria; o__Nitriliruptorales; f__Nitriliruptoraceae; g__Nitriliruptor; s__uncultured_bacterium |
| 253 | 63 | 80 | 80 | k__Bacteria; p__Bacteroidetes; c__Sphingobacteriia; o__Sphingobacteriales; f__Saprospiraceae; g__uncultured |
| 254 | 36 | 45 | 61 | k__Bacteria; p__Bacteroidetes; c__Sphingobacteriia; o__Sphingobacteriales; f__Chitinophagaceae; g__uncultured; s__uncultured_marine_bacterium |
| 255 | 35 | 49 | 52 | k__Bacteria; p__Proteobacteria; c__Alphaproteobacteria; o__Rhodospirillales; f__Acetobacteraceae |
| 256 | 35 | 30 | 36 | k__Bacteria; p__Bacteroidetes; c__Sphingobacteriia; o__Sphingobacteriales; f__Saprospiraceae; g__uncultured |
| 257 | 44 | 49 | 47 | k__Bacteria; p__Bacteroidetes; c__Sphingobacteriia; o__Sphingobacteriales; f__NS11_12_marine_group; g__uncultured_bacterium; s__uncultured_bacterium |
| 258 | 33 | 68 | 57 | k__Bacteria; p__Bacteroidetes; c__Sphingobacteriia; o__Sphingobacteriales; f__Saprospiraceae |
| 259 | 18 | 17 | 10 | k__Bacteria; p__Cyanobacteria; c__Chloroplast; o__Euglena_agilis; f__Euglena_agilis; g__Euglena_agilis; s__Euglena_agilis |
| 260 | 12 | 13 | 19 | k__Bacteria; p__Proteobacteria; c__Deltaproteobacteria; o__Bdellovibrionales; f__Bacteriovoracaceae; g__Peredibacter; Ambiguous_taxa |
| 261 | 1 | 1 | 6 | k__Bacteria; p__Proteobacteria; c__Alphaproteobacteria; o__DB1_14 |
| 262 | 12 | 13 | 24 | k__Bacteria; p__Bacteroidetes; c__Sphingobacteriia; o__Sphingobacteriales; f__NS11_12_marine_group; Ambiguous_taxa; Ambiguous_taxa |
| 263 | 53 | 16 | 95 | k__Bacteria; p__Proteobacteria; c__Alphaproteobacteria; o__Rhodobacterales; f__Rhodobacteraceae; g__Nautella; s__uncultured_bacterium |
| 264 | 24 | 43 | 40 | k__Bacteria; p__Bacteroidetes; c__Bacteroidia; o__Bacteroidales; f__Marinilabiaceae; g__Carboxylicivirga |
| 265 | 30 | 42 | 28 | k__Bacteria; p__Proteobacteria; c__Betaproteobacteria; o__Burkholderiales; f__Alcaligenaceae; g__uncultured |
| 266 | 39 | 38 | 48 | k__Bacteria; p__Proteobacteria; c__Alphaproteobacteria; o__Sphingomonadales; f__Sphingomonadaceae |
| 267 | 21 | 30 | 30 | k__Bacteria; p__Proteobacteria; c__Gammaproteobacteria; o__Chromatiales; f__Chromatiaceae; g__Rheinheimera; s__uncultured_Rheinheimera_sp. |
| 268 | 39 | 2 | 0 | k__Bacteria; p__Firmicutes; c__Clostridia; o__Clostridiales; f__Family_XII; g__Fusibacter; s__uncultured_bacterium |
| 269 | 76 | 3 | 59 | k__Bacteria; p__Proteobacteria; c__Gammaproteobacteria; o__Oceanospirillales; f__Oceanospirillaceae; g__Marinobacterium; Ambiguous_taxa |
| 270 | 14 | 24 | 23 | k__Bacteria; p__Proteobacteria; c__Alphaproteobacteria; o__Rhizobiales |
| 271 | 5 | 0 | 7 | k__Bacteria; p__Firmicutes; c__Clostridia; o__Clostridiales |
| 272 | 76 | 92 | 98 | k__Bacteria; p__Bacteroidetes; c__Sphingobacteriia; o__Sphingobacteriales; f__Saprospiraceae; g__Candidatus_Aquirestis; s__uncultured_bacterium |
| 273 | 32 | 39 | 41 | k__Bacteria; p__Bacteroidetes; c__Cytophagia; o__Cytophagales; f__Cyclobacteriaceae; g__Algoriphagus |
| 274 | 2 | 8 | 6 | k__Bacteria; p__Proteobacteria; c__Alphaproteobacteria; o__Rickettsiales; f__Mitochondria; g__uncultured_bacterium; s__uncultured_bacterium |
| 275 | 4 | 2 | 0 | k__Bacteria; p__Fibrobacteres; c__Fibrobacteria; o__Fibrobacterales; f__Fibrobacteraceae; g__uncultured; s__uncultured_Fibrobacteres_bacterium |
| 276 | 5 | 7 | 11 | k__Bacteria; p__Proteobacteria; c__Gammaproteobacteria; o__SZB30; Ambiguous_taxa; Ambiguous_taxa; Ambiguous_taxa |
| 277 | 13 | 14 | 16 | k__Bacteria; p__Proteobacteria; c__Deltaproteobacteria; o__Myxococcales; f__P3OB_42 |
| 278 | 10 | 19 | 25 | k__Bacteria; p__Proteobacteria; c__Deltaproteobacteria; o__Bdellovibrionales; f__Bdellovibrionaceae; g__OM27_clade; s__uncultured_bacterium |
| 279 | 27 | 17 | 15 | k__Bacteria; p__Bacteroidetes; c__Flavobacteriia; o__Flavobacteriales; f__Flavobacteriaceae; g__Flavobacterium |
| 280 | 7 | 7 | 9 | k__Bacteria; p__Proteobacteria; c__Betaproteobacteria; o__Burkholderiales; f__Burkholderiaceae; g__Cupriavidus |
| 281 | 14 | 30 | 24 | k__Bacteria; p__Bacteroidetes; c__Sphingobacteriia; o__Sphingobacteriales; f__Saprospiraceae; g__uncultured |
| 282 | 54 | 63 | 76 | k__Bacteria; p__Proteobacteria; c__Betaproteobacteria; o__Burkholderiales; f__Burkholderiaceae; g__Lautropia; Ambiguous_taxa |
| 283 | 9 | 13 | 17 | k__Bacteria; p__Proteobacteria; c__Alphaproteobacteria; o__SAR11_clade; f__Chesapeake_Delaware_Bay; Ambiguous_taxa; Ambiguous_taxa |
| 284 | 39 | 56 | 50 | k__Bacteria; p__Bacteroidetes; c__Cytophagia; o__Cytophagales; f__Cyclobacteriaceae |
| 285 | 92 | 129 | 106 | k__Bacteria; p__Bacteroidetes |
| 286 | 37 | 55 | 46 | k__Bacteria; p__Cyanobacteria; c__Chloroplast; o__Euglenaria_anabaena; f__Euglenaria_anabaena; g__Euglenaria_anabaena; s__Euglenaria_anabaena |
| 287 | 4 | 0 | 0 | k__Bacteria; p__Proteobacteria; c__Gammaproteobacteria; o__Thiotrichales; f__Thiotrichaceae; g__uncultured |
| 288 | 41 | 46 | 64 | k__Bacteria; p__Proteobacteria; c__Deltaproteobacteria; o__Bdellovibrionales; f__Bdellovibrionaceae; g__Bdellovibrio; s__uncultured_organism |
| 289 | 12 | 12 | 16 | k__Bacteria; p__Bacteroidetes; c__Flavobacteriia; o__Flavobacteriales; f__NS9_marine_group; g__uncultured_Flavobacteriales_bacterium; s__uncultured_Flavobacteriales_bacterium |
| 290 | 65 | 44 | 57 | k__Bacteria; p__Proteobacteria; c__Betaproteobacteria; o__Burkholderiales; f__Alcaligenaceae; g__MWH_UniP1_aquatic_group; s__marine_metagenome |
| 291 | 22 | 7 | 5 | k__Bacteria; p__Proteobacteria; c__Gammaproteobacteria; o__Xanthomonadales; f__JTB255_marine_benthic_group |
| 292 | 17 | 25 | 24 | k__Bacteria; p__Proteobacteria; c__Alphaproteobacteria; o__Caulobacterales; f__Caulobacteraceae; g__Brevundimonas; Ambiguous_taxa |
| 293 | 2 | 0 | 0 | k__Bacteria; p__Actinobacteria; c__Acidimicrobiia; o__Acidimicrobiales; f__uncultured |
| 294 | 13 | 16 | 11 | k__Bacteria; p__Proteobacteria; c__Alphaproteobacteria; o__Rhodospirillales; f__I_10; g__alpha_proteobacterium_P_4; s__alpha_proteobacterium_P_4 |
| 295 | 10 | 0 | 4 | k__Bacteria; p__Firmicutes; c__Clostridia; o__Clostridiales; f__Family_XII; g__Fusibacter; s__uncultured_bacterium |
| 296 | 21 | 3 | 12 | k__Bacteria; p__Proteobacteria; c__Gammaproteobacteria; o__Vibrionales; f__Vibrionaceae; g__Vibrio; s__Vibrio_vulnificus |
| 297 | 22 | 15 | 26 | k__Bacteria; p__Gemmatimonadetes; c__Gemmatimonadetes; o__Gemmatimonadales; f__Gemmatimonadaceae; g__Gemmatimonas; s__uncultured_bacterium |
| 298 | 10 | 25 | 13 | k__Bacteria; p__Proteobacteria; c__Alphaproteobacteria; o__Caulobacterales; f__Hyphomonadaceae |
| 299 | 33 | 60 | 69 | k__Bacteria; p__Actinobacteria; c__Nitriliruptoria; o__Nitriliruptorales; f__Nitriliruptoraceae; g__Nitriliruptor |
| 300 | 6 | 3 | 1 | k__Bacteria; p__Actinobacteria; c__Acidimicrobiia; o__Acidimicrobiales; f__uncultured |
| 301 | 14 | 15 | 13 | k__Bacteria; p__Proteobacteria; c__Deltaproteobacteria; o__Bdellovibrionales; f__Bdellovibrionaceae; g__OM27_clade |
| 302 | 29 | 33 | 37 | k__Bacteria; p__Proteobacteria; c__Betaproteobacteria |
| 303 | 23 | 27 | 51 | k__Bacteria; p__Actinobacteria; c__Acidimicrobiia; o__Acidimicrobiales; f__Acidimicrobiaceae; g__CL500_29_marine_group; s__uncultured_bacterium |
| 304 | 6 | 2 | 4 | k__Bacteria; p__Bacteroidetes; c__Sphingobacteriia; o__Sphingobacteriales; f__Saprospiraceae; g__Phaeodactylibacter; s__uncultured_bacterium |
| 305 | 21 | 30 | 29 | k__Bacteria; p__Chlorobi; c__Chlorobia; o__Chlorobiales; f__OPB56; g__uncultured_bacterium; s__uncultured_bacterium |
| 306 | 25 | 56 | 37 | k__Bacteria; p__Bacteroidetes |
| 307 | 6 | 8 | 6 | k__Bacteria; p__Bacteroidetes |
| 308 | 13 | 17 | 21 | k__Bacteria; p__Proteobacteria; c__Deltaproteobacteria; o__Oligoflexales; f__Oligoflexaceae |
| 309 | 8 | 2 | 1 | k__Bacteria; p__Proteobacteria; c__Gammaproteobacteria; o__Alteromonadales; f__Alteromonadaceae; g__Marinobacter; s__Marinobacter_hydrocarbonoclasticus |
| 310 | 30 | 28 | 26 | k__Bacteria; p__Bacteroidetes; c__Flavobacteriia; o__Flavobacteriales; f__Flavobacteriaceae; g__NS3a_marine_group; Ambiguous_taxa |
| 311 | 28 | 24 | 36 | k__Bacteria; p__Bacteroidetes; c__Sphingobacteriia; o__Sphingobacteriales; f__NS11_12_marine_group; g__uncultured_bacterium; s__uncultured_bacterium |
| 312 | 18 | 31 | 34 | k__Bacteria; p__Proteobacteria; c__Deltaproteobacteria; o__Bdellovibrionales; f__Bdellovibrionaceae; g__OM27_clade; s__uncultured_bacterium |
| 313 | 7 | 6 | 7 | k__Bacteria; p__Proteobacteria; c__Alphaproteobacteria; o__Rickettsiales |
| 314 | 11 | 9 | 10 | k__Bacteria; p__Proteobacteria; c__Deltaproteobacteria; o__Desulfuromonadales; f__GR_WP33_58; Ambiguous_taxa; Ambiguous_taxa |
| 315 | 11 | 11 | 9 | k__Bacteria; p__Proteobacteria; c__Betaproteobacteria; o__Burkholderiales; f__Comamonadaceae |
| 316 | 9 | 20 | 14 | k__Bacteria; p__Bacteroidetes; c__Sphingobacteriia; o__Sphingobacteriales; f__LiUU_11_161 |
| 317 | 26 | 24 | 22 | k__Bacteria; p__Proteobacteria; c__Alphaproteobacteria; o__Rhodobacterales; f__Rhodobacteraceae |
| 318 | 5 | 3 | 0 | k__Bacteria; p__Cyanobacteria; c__Chloroplast; o__Monoraphidium_neglectum; f__Monoraphidium_neglectum; g__Monoraphidium_neglectum; s__Monoraphidium_neglectum |
| 319 | 7 | 15 | 15 | k__Bacteria; p__Bacteroidetes; c__Cytophagia; o__Order_III; f__uncultured |
| 320 | 57 | 71 | 77 | k__Bacteria; p__Actinobacteria; c__Actinobacteria; o__Frankiales; f__Sporichthyaceae; Ambiguous_taxa; Ambiguous_taxa |
| 321 | 13 | 10 | 22 | k__Bacteria; p__Proteobacteria; c__Alphaproteobacteria; o__Sphingomonadales; f__Sphingomonadaceae; g__Zymomonas; Ambiguous_taxa |
| 322 | 6 | 2 | 3 | k__Bacteria; p__Proteobacteria; c__Alphaproteobacteria; o__Rhizobiales; f__Rhodobiaceae; g__Anderseniella; Ambiguous_taxa |
| 323 | 5 | 8 | 8 | k__Bacteria; p__Cyanobacteria; c__Chloroplast |
| 324 | 15 | 1 | 2 | k__Bacteria; p__Proteobacteria; c__Gammaproteobacteria; o__Oceanospirillales; f__Oceanospirillaceae; g__Neptuniibacter; Ambiguous_taxa |
| 325 | 7 | 17 | 15 | k__Bacteria; p__Proteobacteria; c__Gammaproteobacteria; o__Alteromonadales; f__Alteromonadaceae; g__Salinimonas; Ambiguous_taxa |
| 326 | 19 | 30 | 30 | k__Bacteria; p__Proteobacteria; c__Betaproteobacteria; o__Burkholderiales; f__Comamonadaceae; g__Sphaerotilus |
| 327 | 41 | 0 | 9 | k__Bacteria; p__Firmicutes; c__Clostridia; o__Clostridiales; f__Family_XII; g__Fusibacter |
| 328 | 14 | 13 | 22 | k__Bacteria; p__Gemmatimonadetes; c__Gemmatimonadetes; o__Gemmatimonadales; f__Gemmatimonadaceae; g__uncultured; s__uncultured_bacterium |
| 329 | 25 | 17 | 14 | k__Bacteria; p__Actinobacteria; c__Thermoleophilia; o__Solirubrobacterales; f__480_2 |
| 330 | 9 | 10 | 12 | k__Bacteria; p__Actinobacteria; c__Actinobacteria |
| 331 | 62 | 66 | 72 | k__Bacteria; p__Actinobacteria; c__Acidimicrobiia; o__Acidimicrobiales; f__Acidimicrobiaceae |
| 332 | 29 | 46 | 42 | k__Bacteria; p__Proteobacteria; c__Betaproteobacteria; o__Burkholderiales; f__Burkholderiaceae; g__Limnobacter; s__uncultured_bacterium |
| 333 | 3 | 0 | 0 | k__Bacteria; p__Bacteroidetes; c__Sphingobacteriia; o__Sphingobacteriales; f__Saprospiraceae; g__uncultured |
| 334 | 13 | 40 | 9 | k__Bacteria; p__Proteobacteria; c__Alphaproteobacteria; o__Sphingomonadales; f__Erythrobacteraceae |
| 335 | 4 | 1 | 3 | k__Bacteria; p__Proteobacteria; c__Epsilonproteobacteria; o__Campylobacterales; f__Campylobacteraceae; g__Arcobacter |
| 336 | 27 | 53 | 36 | k__Bacteria; p__Cyanobacteria; c__Chloroplast; o__uncultured_bacterium; f__uncultured_bacterium; g__uncultured_bacterium; s__uncultured_bacterium |
| 337 | 7 | 0 | 6 | k__Bacteria; p__Proteobacteria; c__Gammaproteobacteria; o__Alteromonadales; f__Ferrimonadaceae; g__Ferrimonas; Ambiguous_taxa |
| 338 | 41 | 27 | 44 | k__Bacteria; p__Bacteroidetes; c__Sphingobacteriia; o__Sphingobacteriales; f__NS11_12_marine_group |
| 339 | 18 | 19 | 24 | k__Bacteria; p__Bacteroidetes; c__Flavobacteriia; o__Flavobacteriales |
| 340 | 11 | 0 | 0 | k__Bacteria; p__Proteobacteria; c__Deltaproteobacteria; o__Bdellovibrionales; f__Bacteriovoracaceae; g__Peredibacter |
| 341 | 31 | 42 | 35 | k__Bacteria; p__Actinobacteria; c__Actinobacteria; o__Corynebacteriales; f__Mycobacteriaceae; g__Mycobacterium; s__uncultured_actinobacterium |
| 342 | 9 | 15 | 12 | k__Bacteria; p__Proteobacteria; c__Gammaproteobacteria; o__Cellvibrionales; f__Spongiibacteraceae; g__BD1_7_clade; s__uncultured_bacterium |
| 343 | 2 | 1 | 0 | k__Bacteria; p__Proteobacteria; c__Gammaproteobacteria; o__Oceanospirillales; f__Oceanospirillaceae; g__Amphritea |
| 344 | 19 | 1 | 5 | k__Bacteria; p__Bacteroidetes; c__Bacteroidia; o__Bacteroidales; f__Bacteroidaceae; g__Bacteroides |
| 345 | 29 | 10 | 21 | k__Bacteria; p__Bacteroidetes; c__Flavobacteriia; o__Flavobacteriales; f__Cryomorphaceae |
| 346 | 4 | 0 | 5 | k__Bacteria; p__Bacteroidetes; c__Sphingobacteriia; o__Sphingobacteriales; f__Sphingobacteriaceae; g__Sphingobacterium |
| 347 | 26 | 44 | 36 | k__Bacteria; p__Cyanobacteria; c__Cyanobacteria; o__SubsectionIII; f__FamilyI; g__Romeria_sp._KLL_H_201; s__Romeria_sp._KLL_H_201 |
| 348 | 31 | 34 | 26 | k__Bacteria; p__Bacteroidetes; c__Sphingobacteriia; o__Sphingobacteriales; f__NS11_12_marine_group |
| 349 | 4 | 3 | 6 | k__Bacteria; p__Proteobacteria; c__Deltaproteobacteria; o__Bdellovibrionales; f__Bacteriovoracaceae; g__Peredibacter |
| 350 | 14 | 9 | 7 | k__Bacteria; p__Bacteroidetes |
| 351 | 21 | 10 | 8 | k__Bacteria; p__Actinobacteria; c__Acidimicrobiia; o__Acidimicrobiales; f__Acidimicrobiales_Incertae_Sedis; g__Candidatus_Microthrix; s__uncultured_bacterium |
| 352 | 42 | 45 | 58 | k__Bacteria; p__Bacteroidetes; c__Sphingobacteriia; o__Sphingobacteriales; f__Chitinophagaceae; g__uncultured |
| 353 | 38 | 62 | 72 | k__Bacteria; p__Gemmatimonadetes; c__Gemmatimonadetes; o__Gemmatimonadales; f__Gemmatimonadaceae; g__Gemmatimonas; s__uncultured_Gemmatimonas_sp. |
| 354 | 28 | 47 | 36 | k__Bacteria; p__Cyanobacteria; c__Chloroplast |
| 355 | 5 | 9 | 12 | k__Bacteria; p__Proteobacteria; c__Gammaproteobacteria; o__34P16 |
| 356 | 22 | 35 | 56 | k__Bacteria; p__Bacteroidetes; c__Flavobacteriia; o__Flavobacteriales; f__Flavobacteriaceae; g__Flavobacterium |
| 357 | 4 | 0 | 0 | k__Bacteria; p__Bacteroidetes; c__Flavobacteriia; o__Flavobacteriales; f__Cryomorphaceae; g__Fluviicola |
| 358 | 5 | 8 | 4 | k__Bacteria; p__Proteobacteria; c__Alphaproteobacteria; o__Rhodobacterales; f__Rhodobacteraceae; g__Labrenzia; Ambiguous_taxa |
| 359 | 14 | 19 | 6 | k__Bacteria; p__Bacteroidetes; c__Flavobacteriia; o__Flavobacteriales; f__Cryomorphaceae; g__Fluviicola |
| 360 | 27 | 39 | 26 | k__Bacteria; p__Proteobacteria; c__Gammaproteobacteria; o__Pseudomonadales; f__Moraxellaceae; g__Acinetobacter |
| 361 | 14 | 13 | 9 | k__Bacteria; p__Proteobacteria; c__Alphaproteobacteria; o__Rhodospirillales; f__Rhodospirillaceae |
| 362 | 2 | 0 | 4 | k__Bacteria; p__Bacteroidetes; c__Cytophagia; o__Cytophagales |
| 363 | 13 | 10 | 16 | k__Bacteria; p__Bacteroidetes; c__Sphingobacteriia; o__Sphingobacteriales |
| 364 | 11 | 1 | 1 | k__Bacteria; p__Bacteroidetes; c__Flavobacteriia; o__Flavobacteriales; f__Cryomorphaceae; g__Lishizhenia; s__Lishizhenia_tianjinensis |
| 365 | 24 | 22 | 21 | k__Bacteria; p__Proteobacteria; c__TA18 |
| 366 | 18 | 20 | 25 | k__Bacteria; p__Bacteroidetes; c__Flavobacteriia; o__Flavobacteriales; f__NS9_marine_group; g__uncultured_Bacteroidetes_bacterium; s__uncultured_Bacteroidetes_bacterium |
| 367 | 11 | 0 | 2 | k__Bacteria; p__Proteobacteria; c__Alphaproteobacteria; o__Rhodospirillales; f__Rhodospirillaceae; g__Thalassospira |
| 368 | 39 | 30 | 53 | k__Bacteria; p__Proteobacteria; c__Gammaproteobacteria; o__Oceanospirillales; f__Oceanospirillaceae; g__Pseudospirillum |
| 369 | 30 | 20 | 28 | k__Bacteria; p__Bacteroidetes; c__Sphingobacteriia; o__Sphingobacteriales; f__Saprospiraceae; g__Haliscomenobacter; s__uncultured_bacterium |
| 370 | 10 | 18 | 16 | k__Bacteria; p__Proteobacteria; c__Gammaproteobacteria; o__Xanthomonadales; f__Xanthomonadaceae; g__Aquimonas; Ambiguous_taxa |
| 371 | 12 | 13 | 13 | k__Bacteria; p__Proteobacteria; c__TA18; o__uncultured_bacterium; f__uncultured_bacterium; g__uncultured_bacterium; s__uncultured_bacterium |
| 372 | 15 | 10 | 10 | k__Bacteria; p__Proteobacteria; c__Alphaproteobacteria; o__Rhodospirillales; f__Acetobacteraceae; g__Stella; Ambiguous_taxa |
| 373 | 3 | 1 | 1 | k__Bacteria; p__Proteobacteria; c__Gammaproteobacteria; o__Oceanospirillales; f__Oceanospirillaceae; g__Marinobacterium |
| 374 | 9 | 5 | 8 | k__Bacteria; p__Proteobacteria; c__Deltaproteobacteria; o__Desulfuromonadales; f__GR_WP33_58; g__uncultured_Desulfuromonadales_bacterium; s__uncultured_Desulfuromonadales_bacterium |
| 375 | 10 | 0 | 0 | k__Bacteria; p__Bacteroidetes; c__Bacteroidia; o__Bacteroidales |
| 376 | 26 | 29 | 18 | k__Bacteria; p__Firmicutes; c__Erysipelotrichia; o__Erysipelotrichales; f__Erysipelotrichaceae; g__Erysipelotrichaceae_UCG_004; s__uncultured_bacterium |
| 377 | 46 | 64 | 58 | k__Bacteria; p__Proteobacteria; c__Betaproteobacteria; o__Burkholderiales; f__Comamonadaceae; g__Limnohabitans; Ambiguous_taxa |
| 378 | 1 | 6 | 1 | k__Bacteria; p__Proteobacteria; c__Betaproteobacteria; o__Burkholderiales; f__Comamonadaceae; g__Comamonas; Ambiguous_taxa |
| 379 | 6 | 0 | 0 | k__Bacteria; p__Tenericutes; c__Mollicutes; o__Mycoplasmatales; f__Mycoplasmataceae; g__uncultured; s__uncultured_bacterium |
| 380 | 2 | 0 | 1 | k__Bacteria; p__Spirochaetae; c__Spirochaetes; o__Spirochaetales; f__Spirochaetaceae |
| 381 | 9 | 25 | 15 | k__Bacteria; p__Firmicutes; c__Bacilli; o__Lactobacillales; f__Streptococcaceae; g__Lactococcus; s__Lactococcus_lactis |
| 382 | 12 | 11 | 14 | k__Bacteria; p__Actinobacteria; c__Thermoleophilia; o__Gaiellales; f__uncultured |
| 383 | 25 | 27 | 38 | k__Bacteria; p__Bacteroidetes; c__Sphingobacteriia; o__Sphingobacteriales; f__NS11_12_marine_group; g__uncultured_Flavobacteriales_bacterium; s__uncultured_Flavobacteriales_bacterium |
| 384 | 9 | 16 | 8 | k__Bacteria; p__Bacteroidetes; c__Sphingobacteriia; o__Sphingobacteriales; f__NS11_12_marine_group |
| 385 | 15 | 19 | 20 | k__Bacteria; p__Bacteroidetes; c__Sphingobacteriia; o__Sphingobacteriales; f__Saprospiraceae; g__uncultured |
| 386 | 11 | 9 | 9 | k__Bacteria; p__Proteobacteria; c__Gammaproteobacteria; o__Legionellales; f__Legionellaceae; g__Legionella |
| 387 | 6 | 5 | 9 | k__Bacteria; p__Proteobacteria; c__TA18; o__uncultured_bacterium; f__uncultured_bacterium; g__uncultured_bacterium; s__uncultured_bacterium |
| 388 | 5 | 8 | 7 | k__Bacteria; p__Firmicutes; c__Clostridia; o__Clostridiales; f__Ruminococcaceae; g__Faecalibacterium; Ambiguous_taxa |
| 389 | 8 | 10 | 18 | k__Bacteria; p__Proteobacteria; c__Deltaproteobacteria; o__Myxococcales |
| 390 | 46 | 42 | 59 | k__Bacteria; p__Bacteroidetes; c__Sphingobacteriia; o__Sphingobacteriales; f__Chitinophagaceae |
| 391 | 24 | 27 | 40 | k__Bacteria; p__Proteobacteria; c__Betaproteobacteria; o__Burkholderiales; f__Burkholderiaceae; g__Lautropia; Ambiguous_taxa |
| 392 | 11 | 27 | 34 | k__Bacteria; p__Proteobacteria; c__Deltaproteobacteria; o__Bdellovibrionales; f__Bdellovibrionaceae; g__OM27_clade; s__uncultured_bacterium |
| 393 | 6 | 14 | 18 | k__Bacteria; p__Proteobacteria; c__Alphaproteobacteria; o__Rhodobacterales; f__Rhodobacteraceae |
| 394 | 2 | 3 | 3 | k__Bacteria; p__Proteobacteria; c__Alphaproteobacteria |
| 395 | 8 | 14 | 17 | k__Bacteria; p__Proteobacteria; c__Gammaproteobacteria; o__Oceanospirillales; f__Oceanospirillaceae; g__Pseudospirillum; s__uncultured_bacterium |
| 396 | 23 | 37 | 31 | k__Bacteria; p__Bacteroidetes; c__Flavobacteriia; o__Flavobacteriales; f__Cryomorphaceae; g__Fluviicola; s__uncultured_bacterium |
| 397 | 29 | 31 | 20 | k__Bacteria; p__Bacteroidetes; c__Flavobacteriia; o__Flavobacteriales; f__NS9_marine_group; g__uncultured_bacterium; s__uncultured_bacterium |
| 398 | 17 | 17 | 18 | k__Bacteria; p__Fusobacteria; c__Fusobacteriia; o__Fusobacteriales; f__Hados.Sed.Eubac.3; g__uncultured_bacterium; s__uncultured_bacterium |
| 399 | 18 | 26 | 28 | k__Bacteria; p__Fusobacteria; c__Fusobacteriia; o__Fusobacteriales |
| 400 | 19 | 30 | 20 | k__Bacteria; p__Proteobacteria; c__Deltaproteobacteria; o__Bdellovibrionales; f__Bacteriovoracaceae; g__Peredibacter |
| 401 | 21 | 47 | 21 | k__Bacteria; p__Bacteroidetes; c__Flavobacteriia; o__Flavobacteriales; f__Flavobacteriaceae; g__Actibacter; s__uncultured_bacterium |
| 402 | 3 | 0 | 0 | k__Bacteria; p__Firmicutes; c__Bacilli; o__Bacillales |
| 403 | 25 | 21 | 27 | k__Bacteria; p__Bacteroidetes; c__Flavobacteriia; o__Flavobacteriales; f__Flavobacteriaceae; g__Flavobacterium |
| 404 | 4 | 2 | 6 | k__Bacteria; p__Bacteroidetes; c__Sphingobacteriia; o__Sphingobacteriales; f__Chitinophagaceae |
| 405 | 13 | 22 | 16 | k__Bacteria; p__Proteobacteria; c__Deltaproteobacteria; o__Oligoflexales; f__Oligoflexaceae |
| 406 | 11 | 21 | 24 | k__Bacteria; p__Bacteroidetes; c__Sphingobacteriia; o__Sphingobacteriales; f__NS11_12_marine_group |
| 407 | 1 | 1 | 2 | k__Bacteria; p__Proteobacteria; c__Deltaproteobacteria; o__Myxococcales; f__P3OB_42; g__uncultured_soil_bacterium; s__uncultured_soil_bacterium |
| 408 | 4 | 7 | 5 | k__Bacteria; p__Proteobacteria; c__Gammaproteobacteria; o__B38; f__uncultured_bacterium; g__uncultured_bacterium; s__uncultured_bacterium |
| 409 | 3 | 2 | 0 | k__Bacteria; p__Proteobacteria; c__Gammaproteobacteria; o__Legionellales; f__Legionellaceae; g__Legionella |
| 410 | 4 | 2 | 1 | k__Bacteria; p__Proteobacteria; c__Gammaproteobacteria; o__Order_Incertae_Sedis; f__Family_Incertae_Sedis; g__Marinicella; Ambiguous_taxa |
| 411 | 43 | 67 | 64 | k__Bacteria; p__Proteobacteria; c__Betaproteobacteria |
| 412 | 6 | 5 | 3 | k__Bacteria; p__Proteobacteria; c__Gammaproteobacteria; o__NKB5; f__uncultured_bacterium; g__uncultured_bacterium; s__uncultured_bacterium |
| 413 | 11 | 4 | 9 | k__Bacteria; p__Proteobacteria; c__TA18; o__uncultured_bacterium; f__uncultured_bacterium; g__uncultured_bacterium; s__uncultured_bacterium |
| 414 | 8 | 6 | 13 | k__Bacteria; p__Proteobacteria; c__Gammaproteobacteria; o__Xanthomonadales; f__Xanthomonadaceae; g__Arenimonas |
| 415 | 10 | 11 | 16 | k__Bacteria; p__Proteobacteria; c__Deltaproteobacteria; o__Myxococcales; f__uncultured; g__uncultured_bacterium; s__uncultured_bacterium |
| 416 | 21 | 16 | 21 | k__Bacteria; p__Proteobacteria; c__Alphaproteobacteria; o__Rhizobiales |
| 417 | 8 | 2 | 1 | k__Bacteria; p__Proteobacteria; c__Proteobacteria_Incertae_Sedis; o__Unknown_Order; f__Unknown_Family; g__Candidatus_Thiobios; s__uncultured_gamma_proteobacterium |
| 418 | 13 | 29 | 26 | k__Bacteria; p__Proteobacteria; c__Alphaproteobacteria; o__Rhizobiales; f__Rhizobiales_Incertae_Sedis; g__Alsobacter; Ambiguous_taxa |
| 419 | 5 | 4 | 5 | k__Bacteria; p__Bacteroidetes; c__Flavobacteriia; o__Flavobacteriales; f__Flavobacteriaceae; g__Flavobacterium |
| 420 | 21 | 28 | 36 | k__Bacteria; p__Proteobacteria; c__Gammaproteobacteria; o__Chromatiales; f__Chromatiaceae; g__Rheinheimera; Ambiguous_taxa |
| 421 | 1 | 1 | 0 | k__Bacteria; p__Proteobacteria; c__Gammaproteobacteria; o__Cellvibrionales; f__Halieaceae |
| 422 | 20 | 29 | 26 | k__Bacteria; p__Proteobacteria; c__Deltaproteobacteria; o__Bdellovibrionales; f__Bdellovibrionaceae; g__OM27_clade; s__uncultured_bacterium |
| 423 | 10 | 11 | 5 | k__Bacteria; p__Proteobacteria; c__Alphaproteobacteria; o__Rickettsiales; f__Holosporaceae; g__uncultured; s__uncultured_Holosporaceae_bacterium |
| 424 | 21 | 20 | 19 | k__Bacteria; p__Proteobacteria; c__Alphaproteobacteria |
| 425 | 1 | 1 | 1 | k__Bacteria; p__Firmicutes; c__Clostridia; o__Clostridiales; f__Clostridiaceae_1 |
| 426 | 11 | 20 | 27 | k__Bacteria; p__Proteobacteria; c__Deltaproteobacteria |
| 427 | 8 | 1 | 7 | k__Bacteria; p__Tenericutes; c__Mollicutes; o__Acholeplasmatales; f__Acholeplasmataceae; g__Acholeplasma |
| 428 | 1 | 1 | 7 | k__Bacteria; p__Bacteroidetes; c__Bacteroidia; o__Bacteroidales; f__Marinilabiaceae; g__uncultured |
| 429 | 20 | 36 | 35 | k__Bacteria; p__Bacteroidetes; c__Flavobacteriia; o__Flavobacteriales; f__Cryomorphaceae; g__Fluviicola; s__uncultured_Flexibacter_sp. |
| 430 | 5 | 7 | 4 | k__Bacteria; p__Cyanobacteria; c__Chloroplast |
| 431 | 4 | 2 | 0 | k__Bacteria; p__Bacteroidetes; c__Sphingobacteriia; o__Sphingobacteriales; f__WCHB1_69; g__uncultured_bacterium; s__uncultured_bacterium |
| 432 | 12 | 25 | 22 | k__Bacteria; p__Proteobacteria; c__TA18 |
| 433 | 3 | 7 | 5 | k__Bacteria; p__Bacteroidetes; c__Sphingobacteriia; o__Sphingobacteriales |
| 434 | 11 | 25 | 32 | k__Bacteria; p__Bacteroidetes; c__Sphingobacteriia; o__Sphingobacteriales; f__Saprospiraceae; g__uncultured |
| 435 | 9 | 18 | 11 | k__Bacteria; p__Proteobacteria; c__Deltaproteobacteria; o__Myxococcales; f__mle1_27; Ambiguous_taxa; Ambiguous_taxa |
| 436 | 20 | 44 | 35 | k__Bacteria; p__Proteobacteria; c__Alphaproteobacteria; o__Rhodospirillales; f__Acetobacteraceae; g__Roseomonas |
| 437 | 0 | 1 | 3 | k__Bacteria; p__Proteobacteria; c__Deltaproteobacteria; o__Bdellovibrionales; f__Bdellovibrionaceae; g__OM27_clade; s__uncultured_bacterium |
| 438 | 11 | 12 | 9 | k__Bacteria; p__Bacteroidetes; c__Sphingobacteriia; o__Sphingobacteriales; f__env.OPS_17; g__uncultured_Bacteroidetes_bacterium; s__uncultured_Bacteroidetes_bacterium |
| 439 | 37 | 49 | 41 | k__Bacteria; p__Bacteroidetes; c__Cytophagia; o__Cytophagales; f__Cyclobacteriaceae |
| 440 | 11 | 12 | 4 | k__Bacteria; p__Firmicutes; c__Bacilli; o__Bacillales; f__Paenibacillaceae; g__Paenibacillus |
| 441 | 0 | 3 | 0 | k__Bacteria; p__TA06 |
| 442 | 66 | 73 | 97 | k__Bacteria; p__Proteobacteria; c__Betaproteobacteria; o__Burkholderiales; f__Alcaligenaceae; g__MWH_UniP1_aquatic_group |
| 443 | 14 | 21 | 22 | k__Bacteria; p__Bacteroidetes; c__Flavobacteriia; o__Flavobacteriales; f__NS9_marine_group; g__uncultured_bacterium; s__uncultured_bacterium |
| 444 | 7 | 8 | 6 | k__Bacteria; p__Actinobacteria; c__Actinobacteria; o__Frankiales; f__Sporichthyaceae |
| 445 | 14 | 24 | 24 | k__Bacteria; p__Actinobacteria; c__Actinobacteria |
| 446 | 6 | 6 | 7 | k__Bacteria; p__Proteobacteria; c__Betaproteobacteria; o__Burkholderiales; f__Alcaligenaceae |
| 447 | 16 | 38 | 36 | k__Bacteria; p__Proteobacteria; c__Alphaproteobacteria; o__Caulobacterales; f__Caulobacteraceae; g__Phenylobacterium; Ambiguous_taxa |
| 448 | 7 | 9 | 9 | k__Bacteria; p__Proteobacteria; c__Deltaproteobacteria; o__Bdellovibrionales; f__Bdellovibrionaceae; g__OM27_clade; s__uncultured_bacterium |
| 449 | 14 | 20 | 21 | k__Bacteria; p__Proteobacteria; c__Alphaproteobacteria; o__Rickettsiales; f__Mitochondria |
| 450 | 11 | 23 | 21 | k__Bacteria; p__Bacteroidetes |
| 451 | 12 | 16 | 17 | k__Bacteria; p__Actinobacteria; c__Actinobacteria; o__Micrococcales; f__Microbacteriaceae |
| 452 | 35 | 48 | 52 | k__Bacteria; p__Proteobacteria; c__Betaproteobacteria; o__Burkholderiales; f__Comamonadaceae |
| 453 | 4 | 5 | 9 | k__Bacteria; p__Proteobacteria; c__Deltaproteobacteria; o__Oligoflexales; f__Oligoflexaceae; g__uncultured_bacterium; s__uncultured_bacterium |
| 454 | 15 | 12 | 6 | k__Bacteria; p__Cyanobacteria; c__Chloroplast |
| 455 | 4 | 3 | 1 | k__Bacteria; p__Proteobacteria; c__Deltaproteobacteria; o__Desulfobacterales; f__Desulfobulbaceae; g__uncultured |
| 456 | 11 | 8 | 13 | k__Bacteria; p__Actinobacteria; c__Thermoleophilia; o__Gaiellales; f__uncultured |
| 457 | 16 | 25 | 18 | k__Bacteria; p__Proteobacteria; c__Gammaproteobacteria; o__Xanthomonadales; f__uncultured |
| 458 | 1 | 2 | 2 | k__Bacteria; p__Proteobacteria; c__Alphaproteobacteria; o__Rickettsiales; f__Holosporaceae; g__Candidatus_Paraholospora; s__Candidatus_Paraholospora_nucleivisitans |
| 459 | 5 | 3 | 8 | k__Bacteria; p__Cyanobacteria; c__Chloroplast; o__Lepocinclis_tripteris; f__Lepocinclis_tripteris; g__Lepocinclis_tripteris; s__Lepocinclis_tripteris |
| 460 | 49 | 29 | 14 | k__Bacteria; p__Bacteroidetes; c__Bacteroidia; o__Bacteroidales; f__Marinilabiaceae; g__Carboxylicivirga; s__uncultured_bacterium |
| 461 | 39 | 47 | 44 | k__Bacteria; p__Proteobacteria; c__Betaproteobacteria; o__Burkholderiales; f__Oxalobacteraceae; g__Paucimonas |
| 462 | 10 | 3 | 7 | k__Bacteria; p__Proteobacteria; c__Deltaproteobacteria; o__Myxococcales; f__Phaselicystidaceae; g__Phaselicystis |
| 463 | 1 | 8 | 0 | k__Bacteria; p__Cyanobacteria; c__Chloroplast |
| 464 | 3 | 4 | 2 | k__Bacteria; p__Bacteroidetes; c__Bacteroidia; o__Bacteroidales; f__Marinilabiaceae; g__Carboxylicivirga |
| 465 | 1 | 2 | 0 | k__Bacteria; p__Firmicutes; c__Clostridia; o__Clostridiales; f__Ruminococcaceae; g__Fastidiosipila |
| 466 | 1 | 6 | 4 | k__Bacteria; p__Bacteroidetes; c__Sphingobacteriia; o__Sphingobacteriales |
| 467 | 2 | 2 | 1 | k__Bacteria; p__Bacteroidetes; c__Bacteroidia; o__Bacteroidales; f__Rikenellaceae; g__Blvii28_wastewater_sludge_group; Ambiguous_taxa |
| 468 | 6 | 3 | 5 | k__Bacteria; p__Proteobacteria; c__Deltaproteobacteria; o__Desulfobacterales; f__Desulfobulbaceae; g__uncultured; s__uncultured_Desulfobulbaceae_bacterium |
| 469 | 12 | 15 | 14 | k__Bacteria; p__Proteobacteria; c__Gammaproteobacteria; o__Chromatiales; f__Chromatiaceae; g__Rheinheimera; Ambiguous_taxa |
| 470 | 1 | 7 | 0 | k__Bacteria; p__Proteobacteria; c__Alphaproteobacteria |
| 471 | 7 | 14 | 14 | k__Bacteria; p__Bacteroidetes; c__Bacteroidia; o__Bacteroidales; f__Bacteroidaceae; g__Bacteroides; s__uncultured_bacterium |
| 472 | 8 | 17 | 20 | k__Bacteria; p__Proteobacteria; c__Gammaproteobacteria; o__Methylococcales; f__Methylococcaceae; g__Methylocaldum; Ambiguous_taxa |
| 473 | 4 | 7 | 4 | k__Bacteria; p__Nitrospirae; c__Nitrospira; o__Nitrospirales; f__Nitrospiraceae; g__Nitrospira; Ambiguous_taxa |
| 474 | 2 | 8 | 2 | k__Bacteria; p__Firmicutes; c__Clostridia; o__Clostridiales; f__Family_XII; g__Fusibacter |
| 475 | 4 | 9 | 16 | k__Bacteria; p__Firmicutes; c__Bacilli; o__Lactobacillales; f__Streptococcaceae; g__Streptococcus; s__Streptococcus_dysgalactiae_subsp._equisimilis |
| 476 | 31 | 37 | 36 | k__Bacteria; p__Proteobacteria; c__Deltaproteobacteria; o__Oligoflexales; f__Oligoflexaceae; g__uncultured_Myxococcales_bacterium; s__uncultured_Myxococcales_bacterium |
| 477 | 0 | 3 | 0 | k__Bacteria; p__Proteobacteria; c__Deltaproteobacteria; o__Bdellovibrionales; f__Bdellovibrionaceae; g__OM27_clade; s__uncultured_bacterium |
| 478 | 0 | 1 | 1 | k__Bacteria; p__Proteobacteria |
| 479 | 0 | 7 | 28 | k__Bacteria; p__Gracilibacteria |
| 480 | 0 | 2 | 0 | k__Bacteria; p__Proteobacteria; c__Alphaproteobacteria; o__Sphingomonadales; f__Sphingomonadaceae; g__Sphingobium; s__uncultured_bacterium |
| 481 | 8 | 3 | 4 | k__Bacteria; p__Bacteroidetes; c__Sphingobacteriia; o__Sphingobacteriales; f__Saprospiraceae; g__Phaeodactylibacter; s__uncultured_bacterium |
| 482 | 10 | 27 | 17 | k__Bacteria; p__Proteobacteria; c__Gammaproteobacteria; o__Xanthomonadales; f__Xanthomonadaceae; g__Stenotrophomonas |
| 483 | 7 | 12 | 23 | k__Bacteria; p__Proteobacteria; c__Gammaproteobacteria; o__Oceanospirillales; f__Halomonadaceae; g__Halomonas; Ambiguous_taxa |
| 484 | 6 | 9 | 12 | k__Bacteria; p__Bacteroidetes; c__Sphingobacteriia; o__Sphingobacteriales; f__Sphingobacteriaceae; g__Pedobacter; Ambiguous_taxa |
| 485 | 14 | 2 | 3 | k__Bacteria; p__Tenericutes; c__Mollicutes; o__NB1_n |
| 486 | 6 | 13 | 10 | k__Bacteria; p__Proteobacteria; c__Alphaproteobacteria; o__Rhodospirillales |
| 487 | 5 | 9 | 7 | k__Bacteria; p__Bacteroidetes; c__Sphingobacteriia; o__Sphingobacteriales |
| 488 | 9 | 10 | 7 | k__Bacteria; p__Proteobacteria; c__Gammaproteobacteria; o__Pseudomonadales; f__Pseudomonadaceae; g__Pseudomonas |
| 489 | 3 | 6 | 8 | k__Bacteria; p__Cyanobacteria; c__Chloroplast; o__Euglena_deses; f__Euglena_deses; g__Euglena_deses; s__Euglena_deses |
| 490 | 0 | 8 | 4 | k__Bacteria; p__Proteobacteria; c__Gammaproteobacteria; o__Chromatiales; f__Halothiobacillaceae; g__Thiovirga; s__uncultured_bacterium |
| 491 | 0 | 4 | 3 | k__Bacteria; p__Proteobacteria; c__Betaproteobacteria; o__Neisseriales; f__Neisseriaceae; g__uncultured; s__bacterium_enrichment_culture_clone_B214(2011) |
| 492 | 21 | 35 | 27 | k__Bacteria; p__Proteobacteria; c__Deltaproteobacteria; o__Bdellovibrionales; f__Bdellovibrionaceae; g__OM27_clade; s__uncultured_bacterium |
| 493 | 14 | 15 | 15 | k__Bacteria; p__Proteobacteria; c__Deltaproteobacteria |
| 494 | 4 | 3 | 8 | k__Bacteria; p__Proteobacteria; c__TA18 |
| 495 | 1 | 1 | 5 | k__Bacteria; p__Proteobacteria; c__Deltaproteobacteria; o__Bdellovibrionales; f__Bdellovibrionaceae; g__Bdellovibrio |
| 496 | 1 | 0 | 4 | k__Bacteria; p__Proteobacteria; c__Betaproteobacteria; o__Rhodocyclales; f__Rhodocyclaceae |
| 497 | 16 | 8 | 16 | k__Bacteria; p__Cyanobacteria; c__Chloroplast; o__Euglena_agilis; f__Euglena_agilis; g__Euglena_agilis; s__Euglena_agilis |
| 498 | 3 | 0 | 7 | k__Bacteria; p__Proteobacteria; c__Alphaproteobacteria; o__Rickettsiales; f__SAR116_clade; Ambiguous_taxa; Ambiguous_taxa |
| 499 | 6 | 0 | 18 | k__Bacteria; p__Proteobacteria; c__Gammaproteobacteria; o__Oceanospirillales; f__Oceanospirillaceae; g__Marinomonas; Ambiguous_taxa |
| 500 | 0 | 1 | 3 | k__Bacteria; p__Proteobacteria; c__Deltaproteobacteria; o__Bdellovibrionales; f__Bacteriovoracaceae |
| 501 | 28 | 45 | 34 | k__Bacteria; p__Proteobacteria; c__Alphaproteobacteria; o__Rhodobacterales; f__Rhodobacteraceae; g__Tabrizicola; s__uncultured_bacterium |
| 502 | 0 | 0 | 3 | k__Bacteria; p__Proteobacteria; c__Betaproteobacteria; o__Nitrosomonadales; f__Nitrosomonadaceae; g__Nitrosomonas; s__uncultured_bacterium |
| 503 | 0 | 0 | 14 | k__Bacteria; p__Gracilibacteria |
| 504 | 8 | 3 | 15 | k__Bacteria; p__Proteobacteria; c__Alphaproteobacteria; o__Rhodobacterales; f__Rhodobacteraceae |
| 505 | 16 | 11 | 22 | k__Bacteria; p__Proteobacteria; c__Betaproteobacteria; o__Burkholderiales |
| 506 | 3 | 2 | 2 | k__Bacteria; p__Proteobacteria; c__Alphaproteobacteria; o__Rhizobiales; f__Hyphomicrobiaceae; g__Hyphomicrobium; s__uncultured_bacterium |
| 507 | 8 | 6 | 15 | k__Bacteria; p__Firmicutes; c__Bacilli; o__Lactobacillales; f__Carnobacteriaceae; g__Carnobacterium; Ambiguous_taxa |
| 508 | 4 | 4 | 10 | k__Bacteria; p__Proteobacteria; c__Betaproteobacteria; o__Burkholderiales; f__Comamonadaceae |
| 509 | 9 | 5 | 11 | k__Bacteria; p__Proteobacteria; c__Deltaproteobacteria; o__Bdellovibrionales; f__Bacteriovoracaceae; g__Peredibacter |
| 510 | 11 | 10 | 8 | k__Bacteria; p__Bacteroidetes; c__Flavobacteriia; o__Flavobacteriales; f__NS9_marine_group; g__uncultured_Flavobacteriales_bacterium; s__uncultured_Flavobacteriales_bacterium |
| 511 | 2 | 3 | 5 | k__Bacteria; p__Proteobacteria; c__Deltaproteobacteria |
| 512 | 10 | 20 | 22 | k__Bacteria; p__Proteobacteria; c__Betaproteobacteria; o__Methylophilales; f__Methylophilaceae; g__uncultured; s__uncultured_bacterium |
| 513 | 3 | 10 | 10 | k__Bacteria; p__Proteobacteria; c__Alphaproteobacteria; o__Rickettsiales; f__Rickettsiales_Incertae_Sedis; g__Candidatus_Captivus |
| 514 | 19 | 15 | 18 | k__Bacteria; p__Bacteroidetes; c__Sphingobacteriia; o__Sphingobacteriales; f__Chitinophagaceae; g__uncultured |
