# Supplementary material for: Microbial origin of bioflocculation components within a promising natural bioflocculant resource of Ruditapes philippinarum conglutination mud from an aquaculture farm in Zhoushan, China
Source: PLoS One. 2019 Jun 19;14(6):e0217679. doi: 10.1371/journal.pone.0217679 (PMC6583956; doi:10.1371/journal.pone.0217679)
Supplement: S2 Table — (DOCX) [file pone.0217679.s003.docx]

**S2 Table. Phylogenetic identification of 14 bioflocculant-producing isolates based on 16S rDNA sequences and EzBioCloud's database**

| Strain | Identification | Identity |
| --- | --- | --- |
| GHF1 | *Pseudoalteromonas undina* | 100.00% |
| GHF2 | *Psychrobacter aquimaris* | 99.84% |
| GHF10 | *Psychrobacter cibarius* | 100.00% |
| GHF11 | *Halomonas taeanensis* | 98.83% |
| GHF12^∗^ | *[Kocuria assamensis](https://www.ezbiocloud.net/taxonomy?tn=Kocuria%20assamensis)*  *Kocuria palustris* | 100.00%  100.00% |
| GHF1031 | *Celeribacter baekdonensis* | 99.92% |
| GHF1032 | *Albirhodobacter marinus* | 99.61% |
| GHF1042 | *Halomonas taeanensis* | 98.83% |
| GHF1043 | *Psychrobacter cibarius* | 100.00% |
| GHS5^∗^ | *Pseudoalteromonas distincta*  *Pseudoalteromonas paragorgicola* | 100.00%  100.00% |
| GHS8-1 | *Bacillus aryabhattai* | 100.00% |
| GHS19^∗^ | *Pseudoalteromonas distincta*  *Pseudoalteromonas paragorgicola* | 100.00%  100.00% |
| GHS20^∗^ | *Pseudoalteromonas distincta*  *Pseudoalteromonas paragorgicola* | 99.92%  99.92% |
| GHS21 | *Pseudoalteromonas undina* | 100.00% |

∗ Isolates had two closely related type strains with the highest identity
