# Supplementary material for: Microbial origin of bioflocculation components within a promising natural bioflocculant resource of Ruditapes philippinarum conglutination mud from an aquaculture farm in Zhoushan, China
Source: PLoS One. 2019 Jun 19;14(6):e0217679. doi: 10.1371/journal.pone.0217679 (PMC6583956; doi:10.1371/journal.pone.0217679)
Supplement: S3 Table — (DOCX) [file pone.0217679.s004.docx]

**S3 Table. Monosaccharide composition of EPS from bioflocculant-producing bacterial isolates**

**(Content in %)**

| Monosaccharide composition | GHF1 | GHF21 | GHS5 | GHS19 | GHS20 | GHF1042 | GHF11 | GHF10 | GHF1043 | GHF1031 | GHS8-1 |
| --- | --- | --- | --- | --- | --- | --- | --- | --- | --- | --- | --- |
| Man | 26.13% | 36.63% | 49.95% | 4.89% | 3.97% | 52.36% | 44.11% | 22.83% | 35.29% | 30.49% | 12.92% |
| GlcN | 2.88% | 0.73% | 1.50% | 9.53% | 6.51% | 8.38% | 28.23% | 5.71% | 3.88% | 4.88% | 8.14% |
| Rib | 5.75% | 15.38% | 8.99% | 1.66% | 1.90% | 4.19% | 0.0009% | 5.48% | 1.05% |  | 2.33% |
| Rha | 0.25% | 0.14% |  |  |  |  |  | 1.14% |  | 5.18% |  |
| GlcUA |  |  | 3.96% | 0.73% | 0.48% | 0.52% | 0.35% |  |  | 1.83% | 7.62% |
| GalUA | 0.23% | 0.18% | 0.30% | 0.54% | 0.56% | 18.32% | 7.06% | 0.91% |  | 6.40% |  |
| GalN |  |  | 0.30% | 0.24% | 0.32% | 1.04% | 0.71% | 0.68% |  |  | 0.39% |
| Glc | 50.13% | 43.59% | 30.97% | 66.23% | 72.75% | 11.52% | 18.09% | 37.90% | 54.69% | 34.45% | 51.95% |
| Gal | 3.78% | 2.20% | 1.50% | 8.46% | 7.34% | 1.05% | 0.75% | 24.66% | 2.82% | 14.94% | 10.34% |
| Xyl | 2.08% | 0.26% |  |  |  | 0.52% | 0.71% | 0.23% | 1.41% |  | 0.10% |
| Ara | 0.005% | 0.15% | 0.0005% | 0.04% | 0.40% |  |  | 0.46% | 0.85% | 0.91% |  |
| Fuc | 8.76% | 0.73% | 2.50% | 7.67% | 5.79% | 2.09% |  |  |  | 0.91% | 6.20% |
